# Ultra-broadband on-chip twisted light emitter for optical communications

**DOI:** 10.1038/lsa.2018.1

**Published:** 2018-04-20

**Authors:** Zhenwei Xie, Ting Lei, Fan Li, Haodong Qiu, Zecen Zhang, Hong Wang, Changjun Min, Luping Du, Zhaohui Li, Xiaocong Yuan

**Affiliations:** 1 Nanophotonics Research Center, Shenzhen Key Laboratory of Micro-scale Optical Information Technology, Shenzhen University, Shenzhen 518060, China; 2 State Key Laboratory of Optoelectronic Materials and Technologies and School of Electronics and Information Technology, Sun Yat-sen University, Guangzhou 510275, China; 3 School of Electrical and Electronic Engineering, Nanyang Technological University, Singapore 639798, Singapore

## Abstract

On-chip twisted light emitters are essential components of orbital angular momentum (OAM) communication devices^1, 2^. These devices address the growing demand for high-capacity communication systems by providing an additional degree of freedom for wavelength/frequency division multiplexing (WDM/FDM). Although whispering-gallery-mode-enabled OAM emitters have been shown to possess some advantages^3, 4, 5^, such as compactness and phase accuracy, their inherent narrow bandwidths prevent them from being compatible with WDM/FDM techniques. Here, we demonstrate an ultra-broadband multiplexed OAM emitter that utilizes a novel joint path-resonance phase control concept. The emitter has a micron-sized radius and nanometer-sized features. Coaxial OAM beams are emitted across the entire telecommunication band from 1,450 to 1,650 nm. We applied the emitter to an OAM communication with a data rate of 1.2 Tbit/s assisted by 30-channel optical frequency combs (OFCs). The emitter provides a new solution to further increase capacity in the OFC communication scenario.

Twisted light with a helical wavefront carries OAM^6^; it has a phase profile of exp(*imφ*), where m is the topological charge indicating the mode order of the OAM, and *φ* is the azimuthal angle^7^. OAM beams are widely used in numerous applications, including particle trapping ^8, 9^, quantum memory ^10^, on-chip optical encoding^11^, optical metrology, and microphotography^12, 13^. Recently, OAM has attracted considerable attention for use in mode division multiplexing in free space and in fiber applications for both classical and quantum communication systems^1, 2, 14, 15, 16, 17^; the infinite number of orthogonal states of OAM provide an additional degree of freedom to the conventional multiplexing techniques^18^. On-chip optical interconnects are increasingly replacing wires owing to significant requirements for massive data-link capacities in high-performance computers and warehouse-scale datacenters. OAM communication via on-chip optical interconnects could provide new opportunities for additional multiplexing to address the need for high-capacity, low-power communications technology that is highly compatible with existing WDM/FDM systems. These possibilities have motivated pioneering research into integrated silicon OAM emitters and III-V OAM microlasers based on optical waveguide phase arrays^19, 20, 21^ and whispering-gallery-mode resonators^3, 4, 5^. Waveguide phase arrays have been implemented to generate OAM beams with tunable topological charges^21^, multiple axial OAM beams^19^ and high-order OAM beams^20^ for optical communication. Their main drawback is their relatively large footprint compared with typical silicon photonic devices. For a compact-resonator-based OAM emitter, the intrinsic high quality factor (Q) of the whispering-gallery-mode limits the emission bandwidth and significantly hinders the suitability of these devices for WDM-/FDM-compatible applications^22^. This presents a significant obstacle for future on-chip communication, since on-chip OFC^23, 24, 25, 26, 27^ enabled broadband emitters appear poised to dominate the next generation of multiplexing technologies^28, 29^. Therefore, OAM emitters with broadband capability are required that can convert OFCs to twisted light for further expansion of communication capacity.

In this work, we propose and demonstrate a silicon-based multiplexed OAM emitter with ultra-broad bandwidth in the telecommunication band (from 1450 to 1650 nm); this is achieved via the joint phase control of the optical path and local resonances in subwavelength structures. The working principle is based on the synthesis of a series of nano-sized cavities with low *Q* resonance, enabling accurate phase control as well as short response time. Figure 1a shows a schematic of the multiplexed OAM emitter for broadband input signals with multiple frequencies from *f*_1_ to *f*_*n*_. The emitter has a circular shape containing subwavelength structures and is connected to two single-mode waveguides. OFC signals are input from the left or right side of the waveguide (left or right arm of the device), oscillate in the device, and are emitted vertically into free space in the form of OAM modes with state numbers of −1 or +1, respectively. Therefore, all the frequency channels are simultaneously multiplexed by the OAM through the emitter.

Our OAM emitter was fabricated on a standard silicon-on-insulator wafer with a silicon layer of 220 nm and a buried oxide layer of 2 μm. The radius of the device was chosen to be 1.2 μm; inner resonance structures were around 100 nm in size (Supplementary Section 2). Figure 1b shows the detailed structures and the phase modulation scheme of the OAM emitter. The total phase modulation originates from both the propagation phase delay and the resonance phase delay from the local subwavelength structure (Supplementary Section 1). Thus, the phase modulation for the emitter can be expressed as


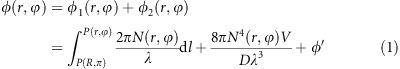


where *r*, *φ* are the polar coordinates (origin is the center of the device), *ϕ* is the phase modulation of the emitter and *ϕ*_*1*_ and *ϕ*_*2*_ are the phase modulations induced by the propagation and localized resonances, respectively. *ϕ*_*1*_ is calculated from the integral along the propagation path, where *P*(*R*, *π*) is the link point for the device and its left arm, *P*(*r*, *φ*) is the local point in the device where the light is emitted out of plane and *N*(*r*, *φ*) is the refractive index distribution of the device determined by the detailed substructures. The estimated propagation delay lies somewhere in the range of 0 to 6*π*, corresponding to a response time <15 fs. *ϕ*_*2*_ is derived with respect to the local refractive index distribution and structure volume, *V* is the volume of the local cavity, *D* is the mode degeneracy and *ϕ′* is the difference between the phase delay and the group delay (between −*π*~π) of the local resonance. The substructures of the designed devices have volumes ranging from 5.9 × 10^−4^λ^3^ to 4.5 × 10^−2^*λ*^3^ (see Supplementary Section 1). The calculated phase delay is sufficient for a 2*π* phase modulation, while the corresponding *Q* of the local cavity is below 15. Therefore, the designed device is equipped with full phase control and ultra-fast time–response properties. According to the time–bandwidth product relationship, the emitter should also have a broadband spectral domain. Noting that light from the left arm yields the *m*_1_ order OAM mode and light from the right arm yields the *m*_2_ order OAM mode, we obtain the following set of Equations:


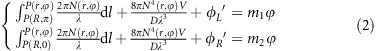


The solutions for *N*(*r*,*φ*) in Equation (2) determine the detailed substructures of the device. However, they cannot be derived analytically. Here, we utilized a global optimization algorithm to solve the equation set (Supplementary Section 2). For the proof-of-concept demonstration, we chose parameters *m*_1_ and *m*_2_ to be +1 and −1 to design a multiplexed emitter for OAM modes with topological orders of +1 and −1. The optimized device with detailed substructures is shown in Figure 1b; the insert shows a scanning electron microscope image of the fabricated device. A device that can emit multiple OAM modes with larger topological charges can be achieved with the proposed method also; one may need to expand the device area and reduce the pixel size to increase the pixel number and to add more freedom in phase control.

To further investigate the device’s high-speed response, we conducted finite-difference time-domain (FDTD) simulations in which we sent a 40 fs signal with a wavelength between 1300 and 1900 nm into the right arm and measured the responses at eight monitor points (see Figure 2a). Points A1 (0.8 μm, *π*/2), B1 (0.8 μm, 3*π*/2), C1 (0.8 μm, *π*) and D1 (0.8 μm, 0) were all in the plane of the device, while points A2, B2, C2 and D2 correspond to A1, B1, C1 and D1 but were shifted 600 nm upwards. Since A2 and B2 as well as C2 and D2 are centrally symmetric points in the emitted OAM state of 1, the phase difference between them should be *π*. From the simulated time-domain profiles (Figure 2b) we deduced that the time responses for points A1 and B1 were almost synchronized, which indicates that the propagation delays for these two points were almost the same. Because of the local resonance, the phase difference between the fields radiated from points A2 and B2 was *π* (±5%) from 38 to 55 fs, as predicted for the OAM state of 1. In this case, the majority of the contribution to the overall phase was from the local resonance. For points C1 and D1, the propagation time delay difference was ∼15 fs. The phase difference between points C2 and D2 was adjusted to *π* (±5%) from 45 to 65 fs via the local resonance. The total phase difference consisted of both propagating phase and resonance phase contributions. Figure 2c and 2d show the FDTD simulated time response of the emitted OAM modes with state numbers of +1 and −1 at locations 600 nm above the surface. The total time response was <40 fs for the OAM emissions. These results verify not only the design principle but also the ultra-fast response of the proposed device.

In Figure 3a, the red and blue solid lines show the FDTD simulation for the transmission efficiency for the OAM emitter in the wavelength range from 1300 to 1900 nm. The OAM +1 and −1 modes had 3 dB emission bandwidths that were 412 nm in width (1400–1812 nm) and 447 nm in width (1300–1747 nm), respectively. The red and blue triangles show the measured emission efficiencies of the +1 and −1 modes from 1450 to 1650 nm. The emission bandwidth was characterized using a tunable laser. The experimental setup is illustrated in Figure 3b (measurement details are shown in Supplementary Section 3). From Figure 3a, we can observe that the maximum emission efficiency reached 35% at 1550 nm, with fluctuations <5 dB over the entire wavelength range of 200 nm. Figure 3c shows the FDTD simulation for emissions of the −1 and +1 OAM modes at 1550 nm. The simulated intensity pattern displays a hollow shape, while the phase profiles indicate counterclockwise and clockwise spiral phases. Figure 3d shows the experimentally measured intensity and interference patterns from 1450 to 1650 nm at intervals of 50 nm. The sign of the topological charge is indicated by the chirality of the interference pattern. The measured mode purity ranged from 90% (at 1650 nm) to 97% (at 1550 nm) for the +1 OAM mode and from 84% (at 1650 nm) to 95% (at 1550 nm) for the −1 OAM mode (Supplementary Section 3). Although the intensity profiles demonstrate asymmetry at some wavelengths, the relatively small phase deviations still ensure the high mode purity of the OAM (Supplementary Section 3). This demonstrates that the generated OAM modes possess the desired phase distribution across the entire 200-nm-wide telecommunication band. To verify that the proposed approach can be adapted to a device design for simultaneously emitting a large number of OAM modes, we designed and demonstrated a four-port OAM emitter with FDTD simulations (see Supplementary Information, Section 3.3).

As a proof of concept, we demonstrated the use of the proposed multiplexed OAM emitter for high-capacity communication applications by testing it with a commercial FDM system. The FDM module generated a set of 30 frequency combs with 0.2-nm-wide channel intervals. Each frequency channel was loaded with 20 Gbit s^−1^ quadrature phase shift keying signals; thus, the total data rate for the emitter associated with the two multiplexed OAM modes was 1.2 Tbit s^−1^. Figure 4a shows the measured bit error rate (BER) for the device used as a OAM mode multiplexer at a receive power of −18 dBm (−1 mode) and −17.4 dBm (+1 mode) for all 30 wavelengths, and the insert shows the corresponding signal constellation. From the time-reversal symmetry of Maxwell’s equations, the proposed device can also be used as a demultiplexer of the two OAM modes. The measured BER for the device used as an OAM mode demultiplexer at a receive power of −18.5 dBm (−1 mode) and −18.2 dBm (+1 mode) for all 30 wavelengths is shown in Figure 4b, and the corresponding signal constellation is shown in the insert. For all channels, the BER was below the hard-decision forward-error-correction limit of 3.8 × 10^−3^. The BER curves for the back-to-back test case and the emissions and the detections of the OAM modes under different received powers were also measured (Figure 4c). In Figure 4c, we can observe some received optical power penalties compared to optical back-to-back when the device is applied to either OAM mode generation or detection. The penalties are induced by cross-talk between the two OAM modes. Additionally in Figure 4c, the QPSK signal on both +1 and −1 modes demonstrate better BER performances for mode generation; this is because the device has higher mode conversion efficiency in mode generation.

In conclusion, we demonstrated a multiplexed on-chip OAM emitter with an ultra-broadband response between 1450 and 1650 nm. Emission efficiencies as high as 35% were achieved, and the mode purity exceeded 97%. It is possible to adapt the design for simultaneously generating multiple high-order OAM states by increasing the device area or reducing the pixel size to further increase the pixel number. The bulky high-numerical aperture objective lens used in the current proof-of-concept status can potentially be replaced by a micron-sized collimator on top of the OAM emitter using the 3D direct writing technique^30^. Furthermore, in the future, the generated OAM modes could also be butt-coupled into an OAM fiber (ring core, air core, multiring cores, few mode, etc.) without an objective. Since we utilized standard integrated circuit techniques, the proposed device is suitable for mass production by the microelectronics industry. In conjunction with integrated OFCs^27^, the proposed OAM multiplexed emitter paves the way for large capacity chip-to-chip optical interconnects. Therefore, we believe the proposed device, with superior performance in terms of bandwidth, multiplexing and integration, could play an important role in OAM communication. More importantly, the proposed joint path resonance phase control will inspire more in-depth fundamental research on manipulating and mapping phase structures for general broadband photonic devices.

## Methods

### Fabrication process of the OAM emitter

The photonic integrated circuit was fabricated on a silicon-on-insulator wafer with a 220-nm-thick silicon layer on top of a 2-μm-thick buried oxide layer. The layout patterns were defined using a 100 kV electron-beam lithography system (Raith EBPG 5200), while a proximity-effect correction was performed using the Layout BEAMER computational software package. A positive e-beam resist (ZEP520A) was used owing to its high dry etch resistance and high resolution. The photonic structures were then transferred to the silicon device layer via a deep reactive-ion etching process, followed by resist removal with dimethylacetamide (see Supplementary Fig. S2 in Supplementary Information).

### Global optimization algorithm

To obtain the solution of Equation (2) in the main text, a global optimization algorithm was proposed and performed. Optimization algorithms are widely utilized for the design of photonic structures. There are several popular algorithms, such as the simulated annealing, genetic, greedy and ant colony algorithms. Recently, an inverse-design algorithm and a direct binary search (DBS) algorithm have been proposed and implemented for nanophotonic design^31, 32^. Theoretically, the inverse design and the DBS should be very suited to in-plane photonic structure design. However, the solution for a mode conversion from in-plane to free space is much more complicated than the in-plane-only mode conversion. To achieve a precise phase modulation, we needed to implement the optimization search in a wider solution space. Because the inverse-design and DBS algorithms tend to find a local optimization instead of a global one, they are more suitable for optimizations in a small solution space. With a large solution space, we needed to introduce the simulated-annealing or the genetic algorithm. Here, we proposed a global optimization method that combines the annealing algorithm and the genetic algorithm associated with three-dimensional FDTD simulations. The idea was to use a random mixture of several best guesses to construct a new guess that could expand the search space without adding too much computational load. Further, to simplify the optimization, we used the inverse process to optimize the structure design; this means that in the optimization process, the device was used to couple the two different OAMs to either the left or the right arm. The figure of merit (FOM) for the optimization was defined as the average coupling efficiency for the two OAM modes. Here, the annealing temperature is defined as the difference between the mode-coupling efficiency of a random status and the target for the OAM emitter. A high initial annealing temperature could guarantee a large search space. However, excessively high initial temperature would markedly increase the computing time. Thus, the initial temperature was set as the possible maximum difference between the mode-coupling efficiency of a random status and the target. The cooling rate also has an effect similar to that of temperature. It is chosen mainly by experience. Here, we set the cooling equation as *Tn*=*T*0=0.98^*n*^, where the cooling rate is 0.98, *T*_0_ is the initial temperature and *T*_*n*_ is the temperature for the *n*th iteration.

The design area was separated into 288 pixels, each of which was either silicon or air (see Supplementary Fig. S1 in Supplementary Information). The specific optimization process was as follows:

1. *Initial guess for the structure design*: The initial temperature was set high enough to ensure a sufficient search space. Since the optimization efficiency is markedly increased by a good initial guess, the initial was a hologram of both OAM modes, which was then transformed into a binary material distribution.
2. *Monitoring the FOM*: If the FOM improved, then the temperature was reduced following the cooling equation, and the best so far and the second best so far values were stored. However, if the FOM worsened, then the second best so far was replaced by exp[(*η*_*n*_−*η*_*n*_−_*1*_)/*T*_*n*_], where *η*_*n*_ is the mode-coupling efficiency of the *n*th guess and *η*_*n−1*_ is the efficiency for the last guess. A new guess was obtained by randomly mixing the best so far and second best so far.
3. Iterate the above process until the FOM reached its maximum or the process exceeded the maximum iteration limit.

## Author contributions

XY and TL developed the concept presented in this paper. ZX conducted the analytical and numerical modeling and designed the device. HQ, ZZ and HW fabricated the device. ZX, TL and FL conducted the measurements. XY, ZL and TL supervised the entire project. ZX, TL, LD and CM wrote the manuscript. All authors discussed the results and commented on the article.

## Figures and Tables

**Figure 1:**
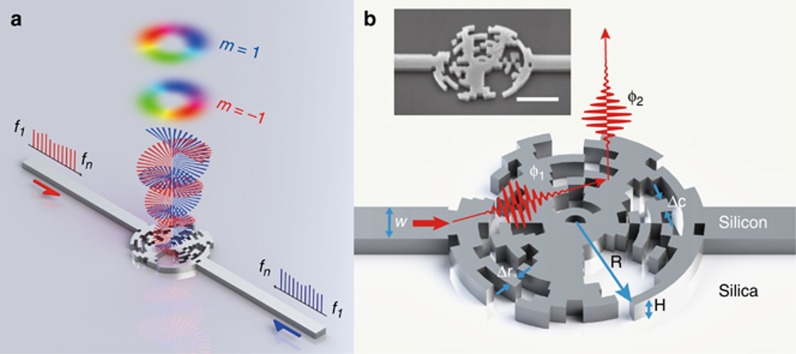
Schematically illustrated principle and structure design of the broadband multiplexed OAM emitter. (**a**) Schematic illustration of the operation of the multiplexed OAM emitter. (**b**) Details of the structure design. *R* denotes the radius of the device (*R*=1.2 μm), *H* the height of the device (*H*=220 nm) and *W* the width of the waveguide (*W*=440 nm). The red arrow indicates light arriving from the left arm. The two red pulses denote the propagating phase *ϕ*_1_ and the resonance phase *ϕ*_2_. The insert shows a scanning electron microscope (SEM) image of the fabricated OAM emitter with a scale bar=1 μm.

**Figure 2:**
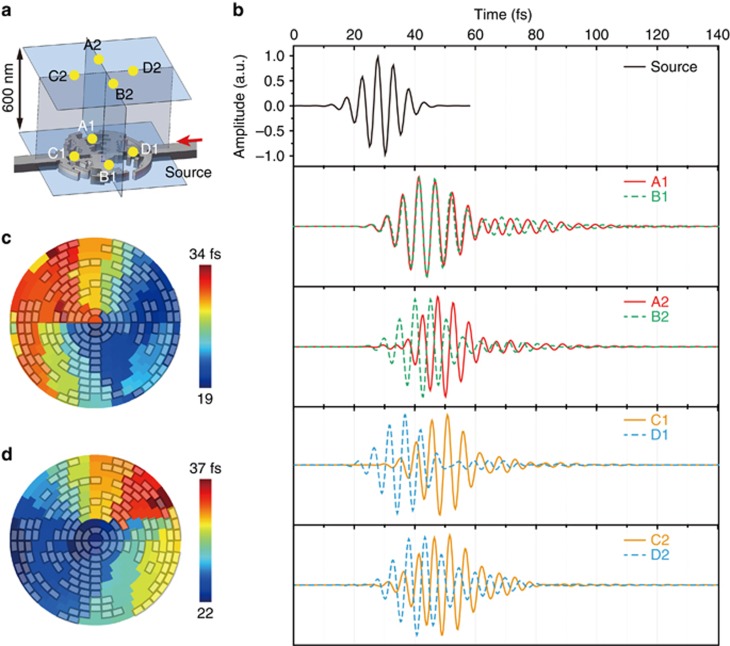
High-speed response of the OAM emitter. (**a**) The locations of the four in-plane monitoring points (A1, B1, C1 and D1) and four out-of-plane monitoring points (A2, B2, C2 and D2, all located 600 nm above the device) for characterizing the OAM emitter time response. (**b**) The FDTD simulation results of the time responses for the eight points detailed in **a** for the OAM mode +1 emission. (**c**, **d)** The time responses of the +1 **c** and −1 **d** OAM modes (group delay), respectively, for each pixel 600 nm above the surface.

**Figure 3:**
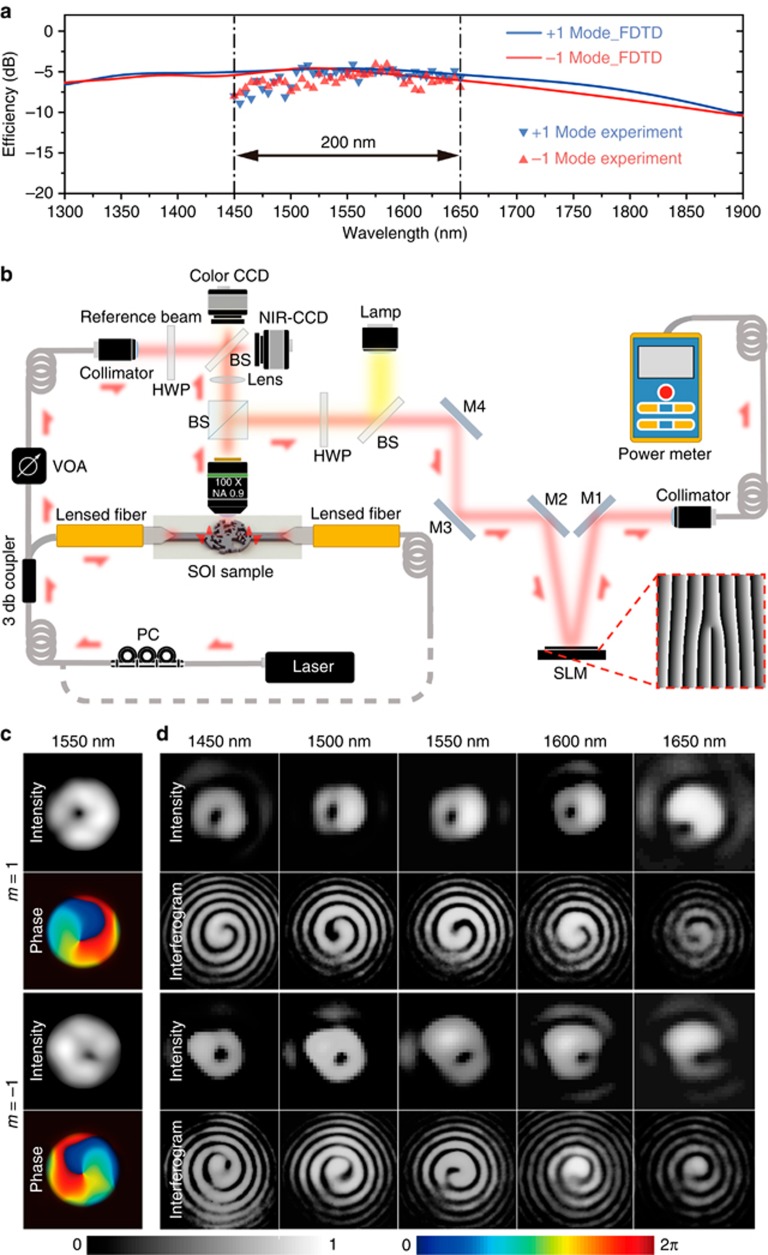
Broadband characteristics of the multiplexed OAM emitter. (**a**) Comparison between the experimental characterization (red and blue triangles) and FDTD simulation results (red and blue lines) for the emission of the OAM modes +1 and −1, respectively, between 1450 and 1650 nm. (**b**) Experimental setup for characterizing the phase and the intensity profiles of the OAM modes generated from the device and for testing its emission bandwidth. VOA: variable optical attenuator; BS: beam splitter; HWP: half wave plate; PC: polarization controller; SLM: spatial light modulator. (**c**) FDTD simulated far-field intensity and phase profiles for the emissions of the −1 and 1 OAM modes at 1550 nm. (**d**) Measured far-field intensity distribution and interference patterns for the emission of the −1 and 1 OAM modes at wavelengths of 1450, 1500, 1550, 1600, and 1650 nm.

**Figure 4:**
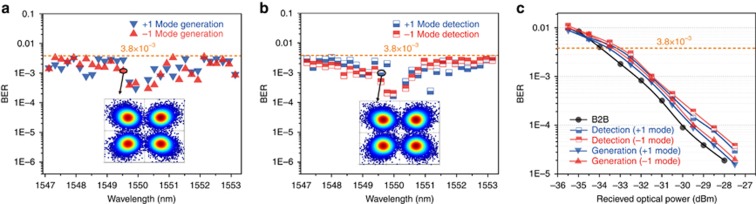
Quadrature phase shift keying communication (de)multiplexing results for 30 frequency combs and two OAM modes. (**a**) The measured bit-error rates (BERs) of the +1 OAM mode (blue triangles) and −1 OAM (red triangles) at total receive powers of −18 dBm (−1 mode) and −17.3 dBm (+1 mode) for all 30 wavelengths. The device is used as an OAM multiplexer. The BERs of all channels were less than the hard-decision forward-error-correction limit of 3.8 × 10^−3^. The insert shows the corresponding signal constellations when the BER was 1.0 × 10^−3^. (**b**) The measured BERs of the +1 OAM mode (blue rectangles) and −1 OAM (red rectangles) at total receive powers of −18.5 dBm (−1 mode) and −18.2 dBm (+1 mode) for all 30 wavelengths. The device was used as an OAM demultiplexer. The BERs of all channels were less than the hard-decision forward-error-correction limit of 3.8 × 10^−3^. The insert shows the corresponding signal constellations when the BER was 1.0 × 10^−3^. (**c**) BER measurements for a back-to-back (B2B) test case, +1 and −1 modes detection and generation at 1550 nm.
