# Supplementary figures and images for: Ultra-broadband on-chip twisted light emitter for optical communications
Source: Light Sci Appl. 2018 Apr 20;7:18001–. doi: 10.1038/lsa.2018.1 (PMC6060059; doi:10.1038/lsa.2018.1)

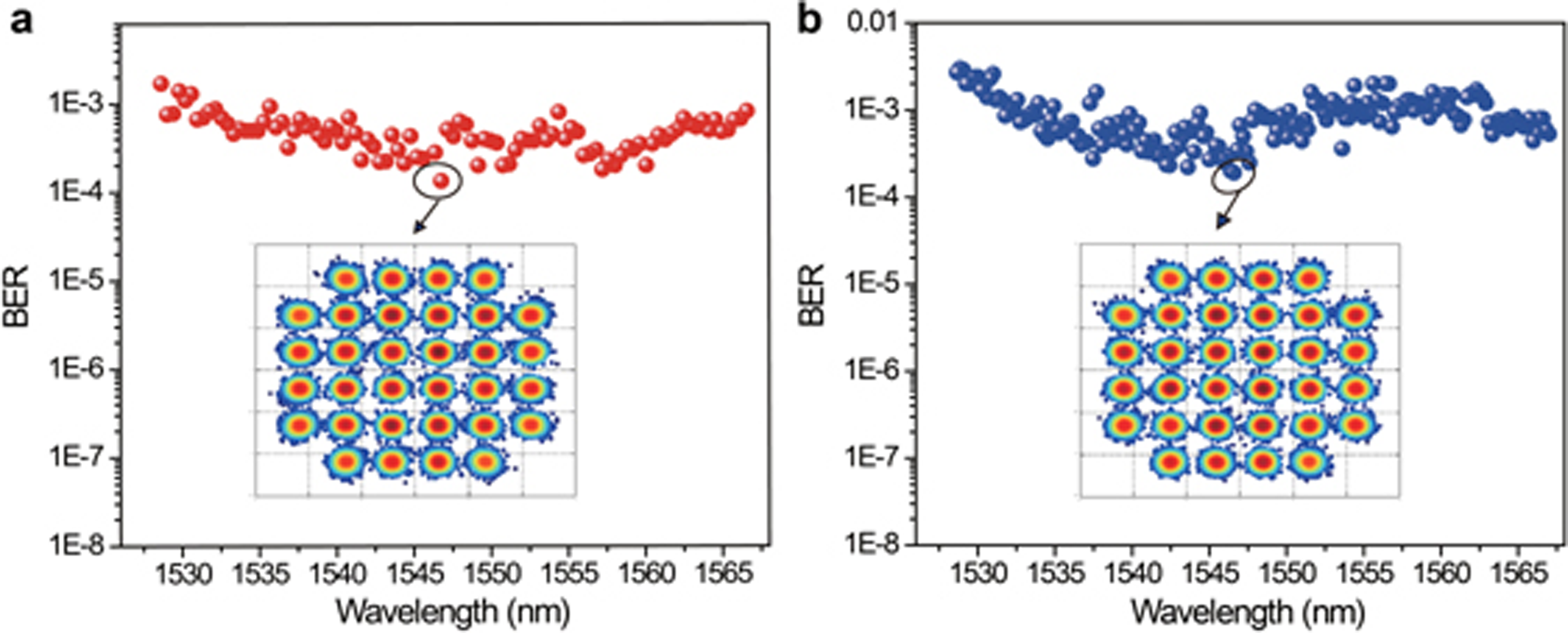

Supplement: Supplementary Figure S1 [file lsa20181x1.tif]

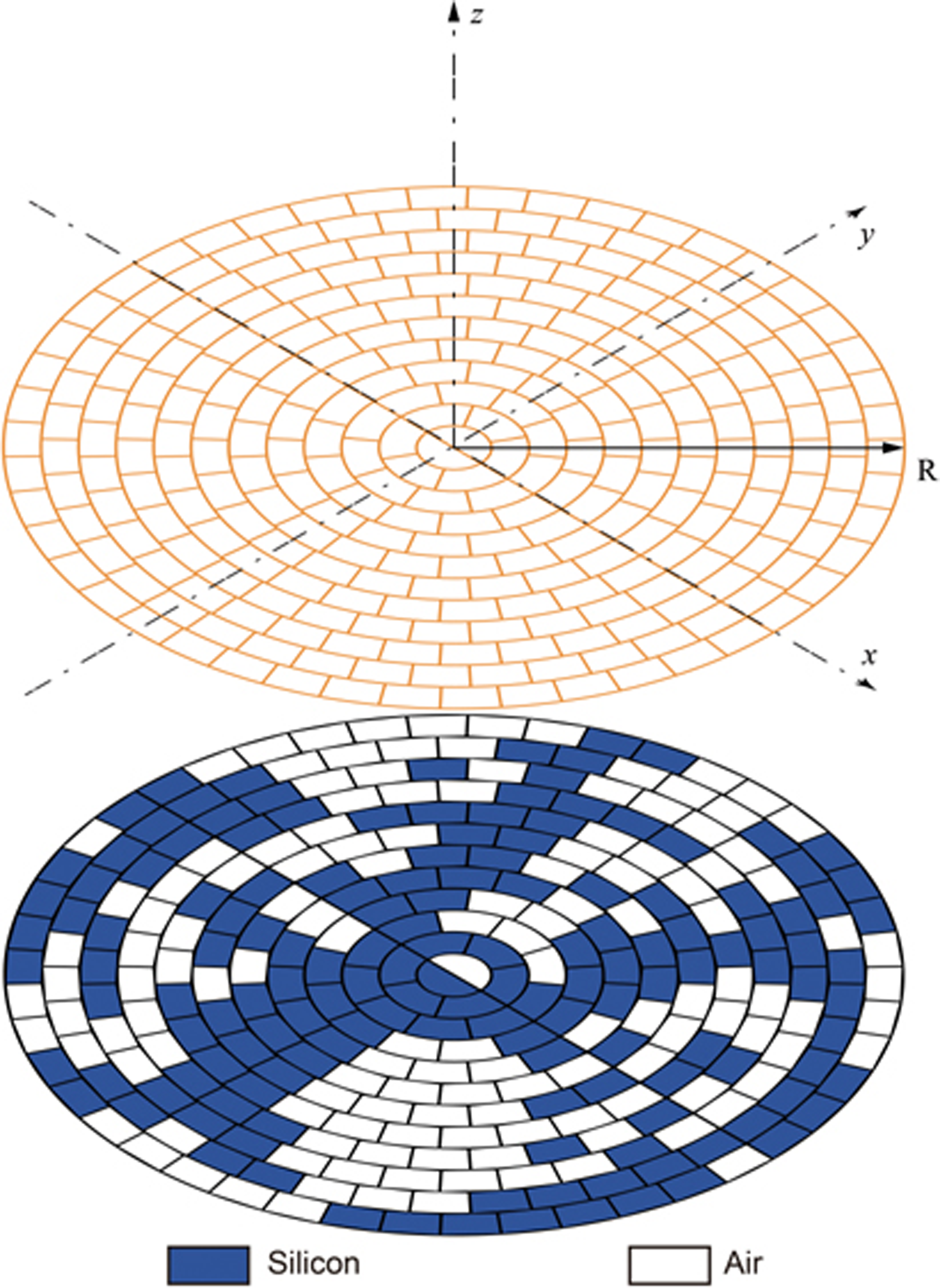

Supplement: Supplementary Figure S2 [file lsa20181x2.tif]

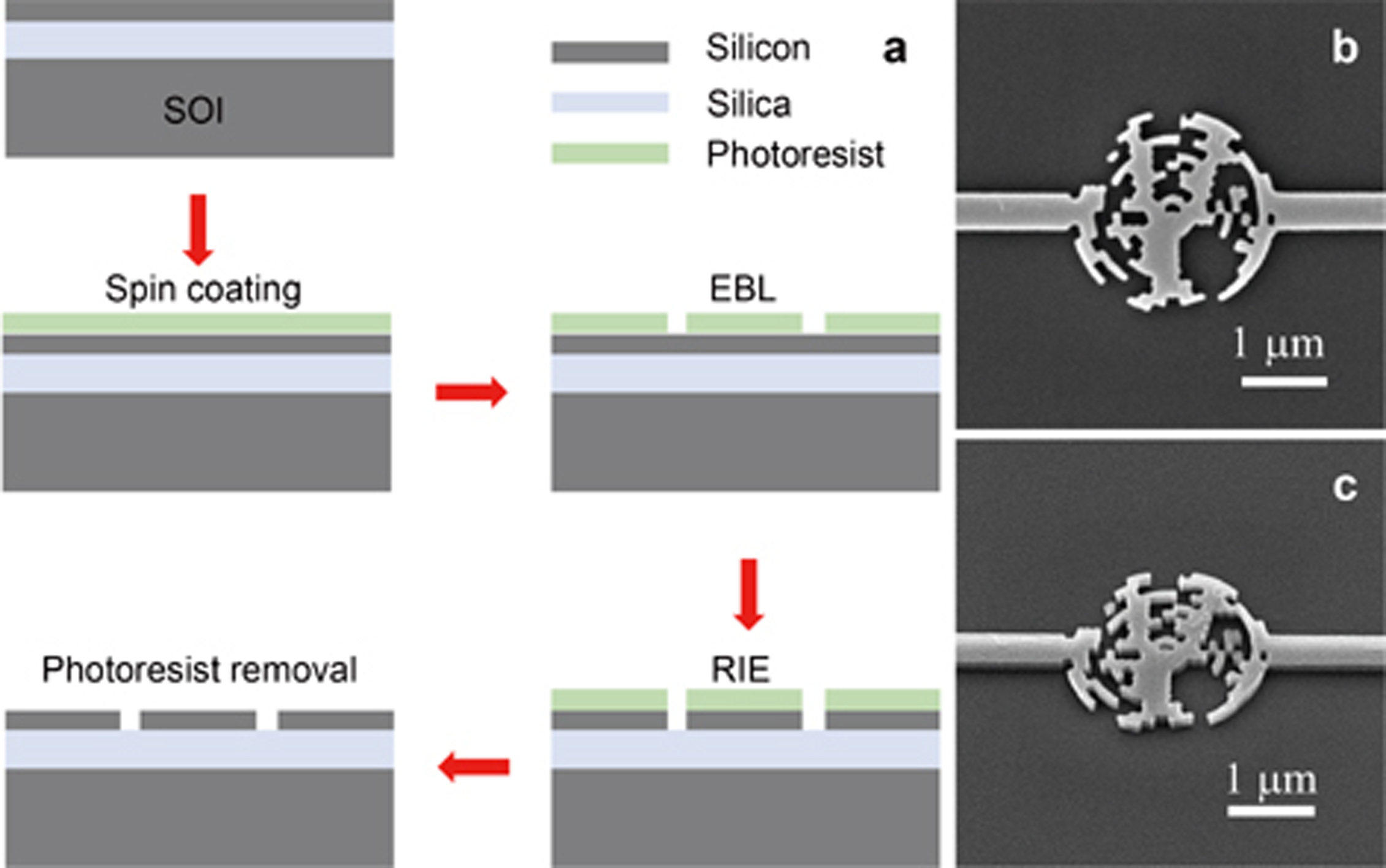

Supplement: Supplementary Figure S3 [file lsa20181x3.tif]

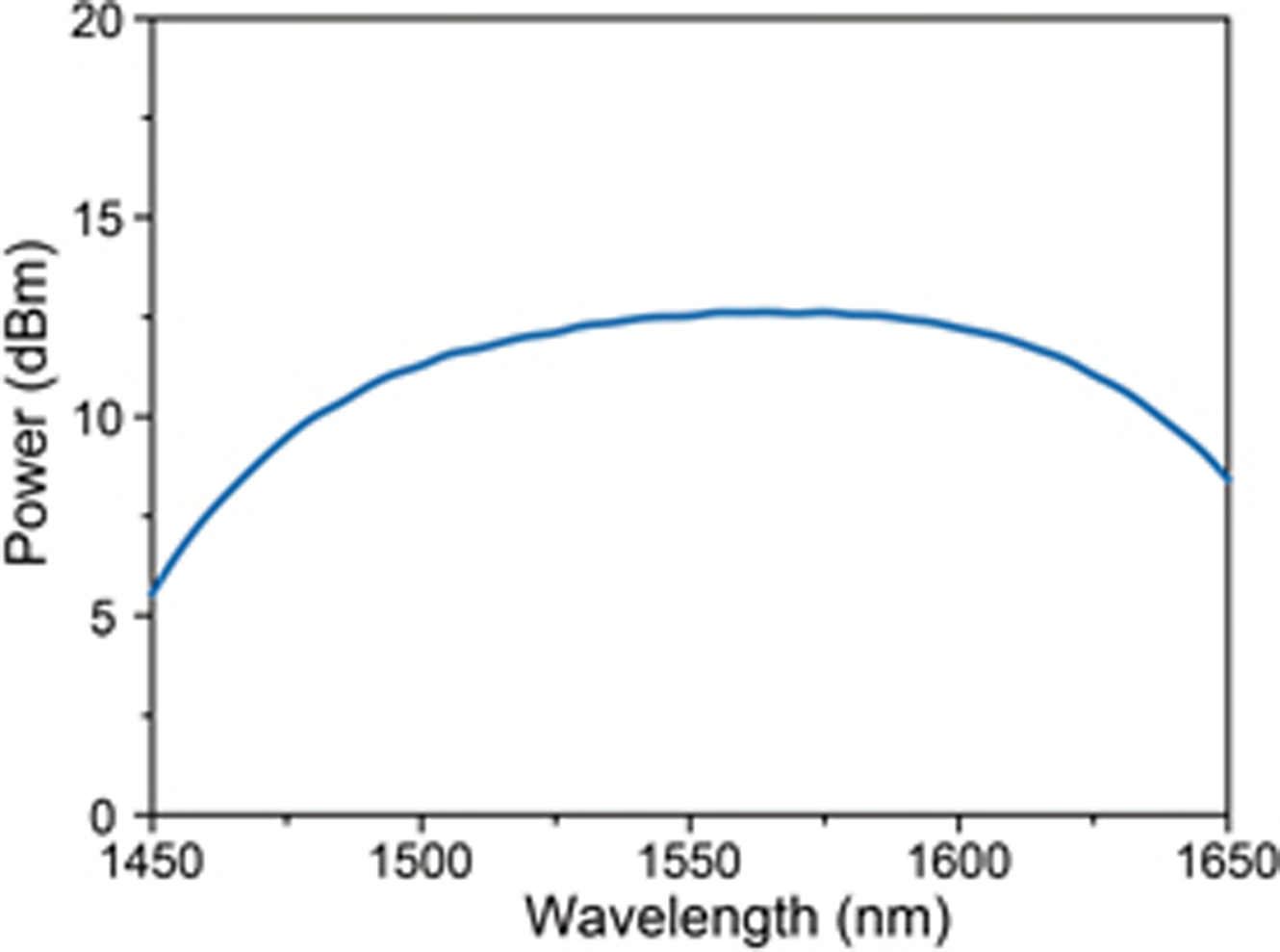

Supplement: Supplementary Figure S4 [file lsa20181x4.tif]

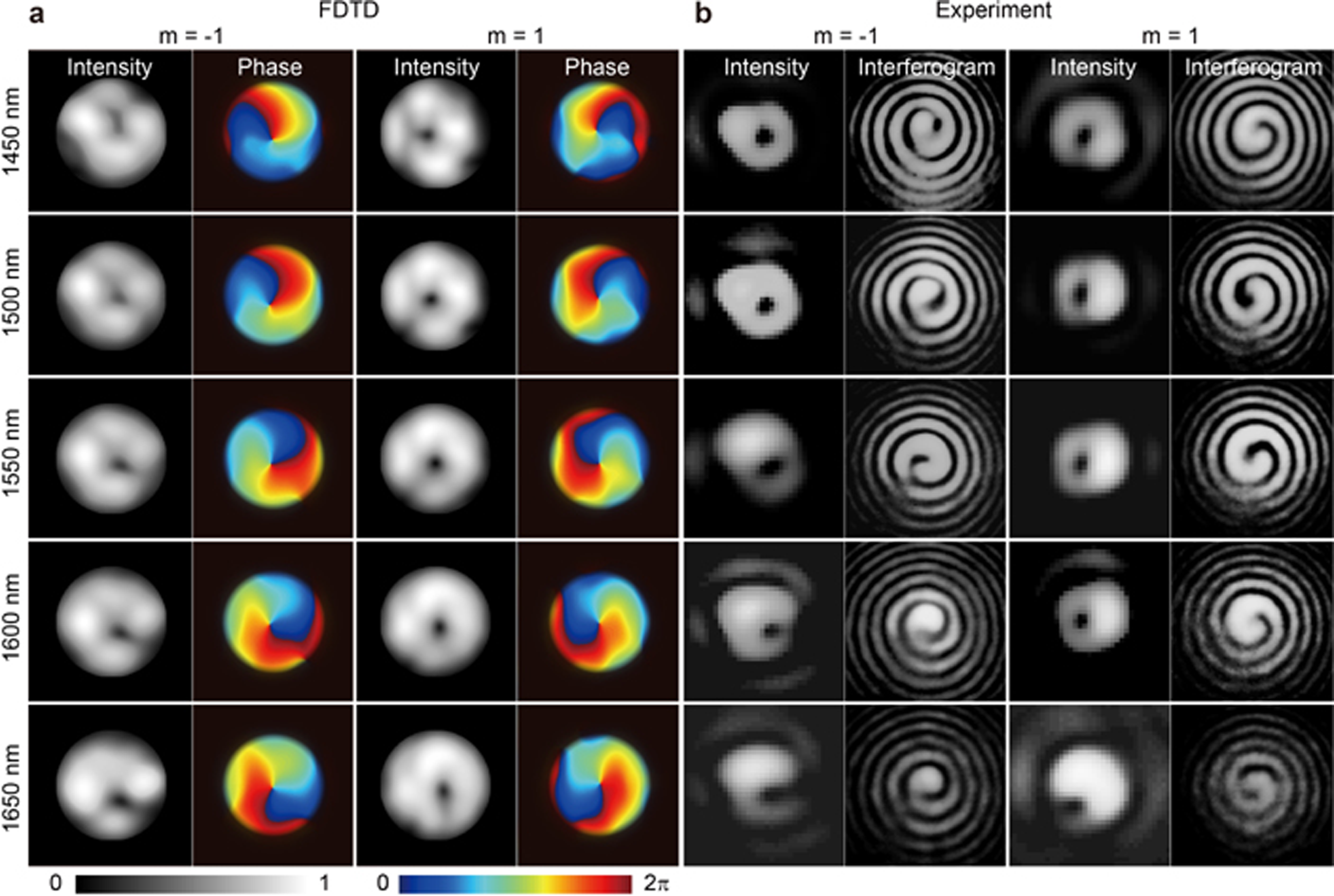

Supplement: Supplementary Figure S5 [file lsa20181x5.tif]

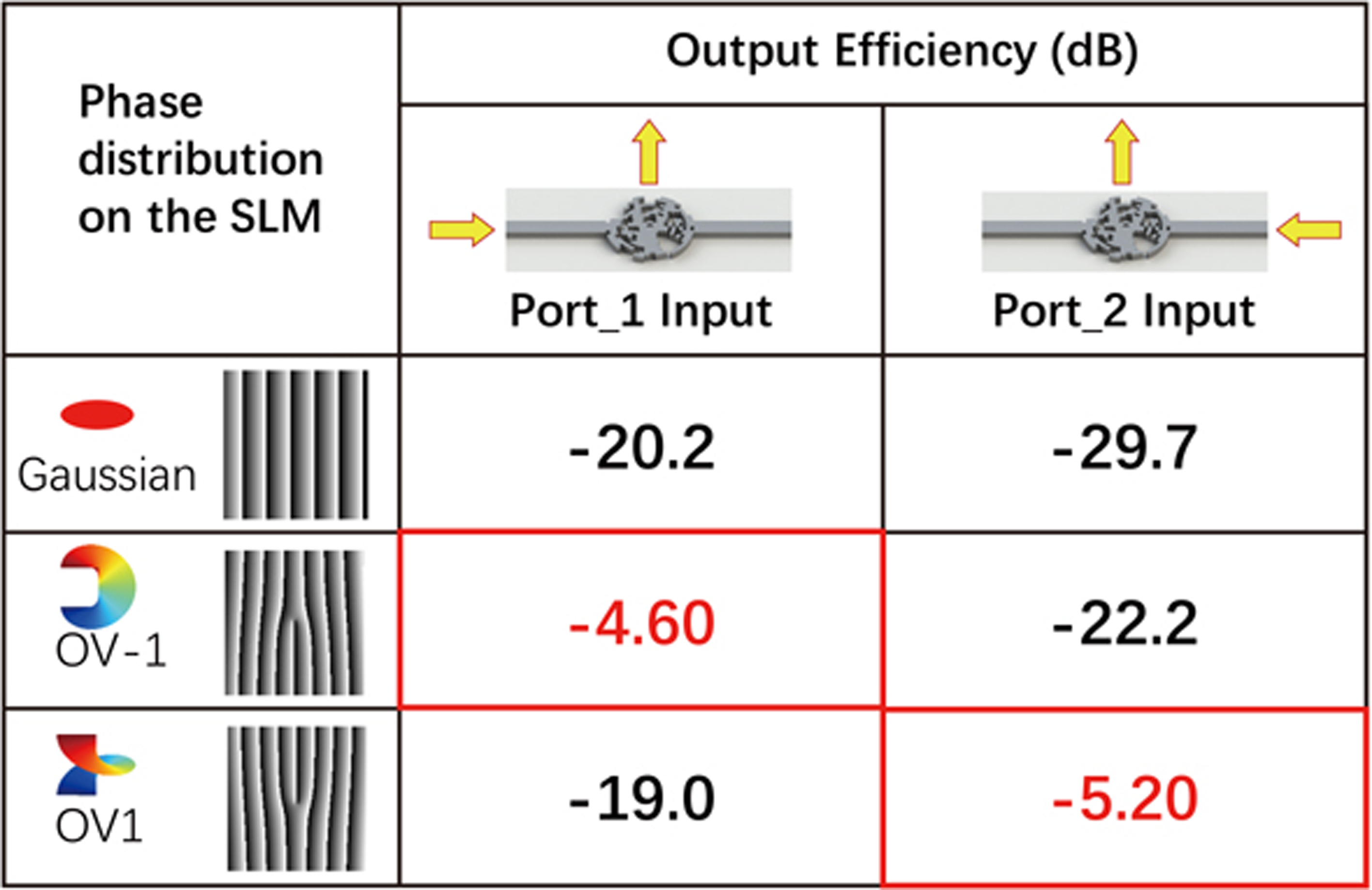

Supplement: Supplementary Figure S6 [file lsa20181x6.tif]

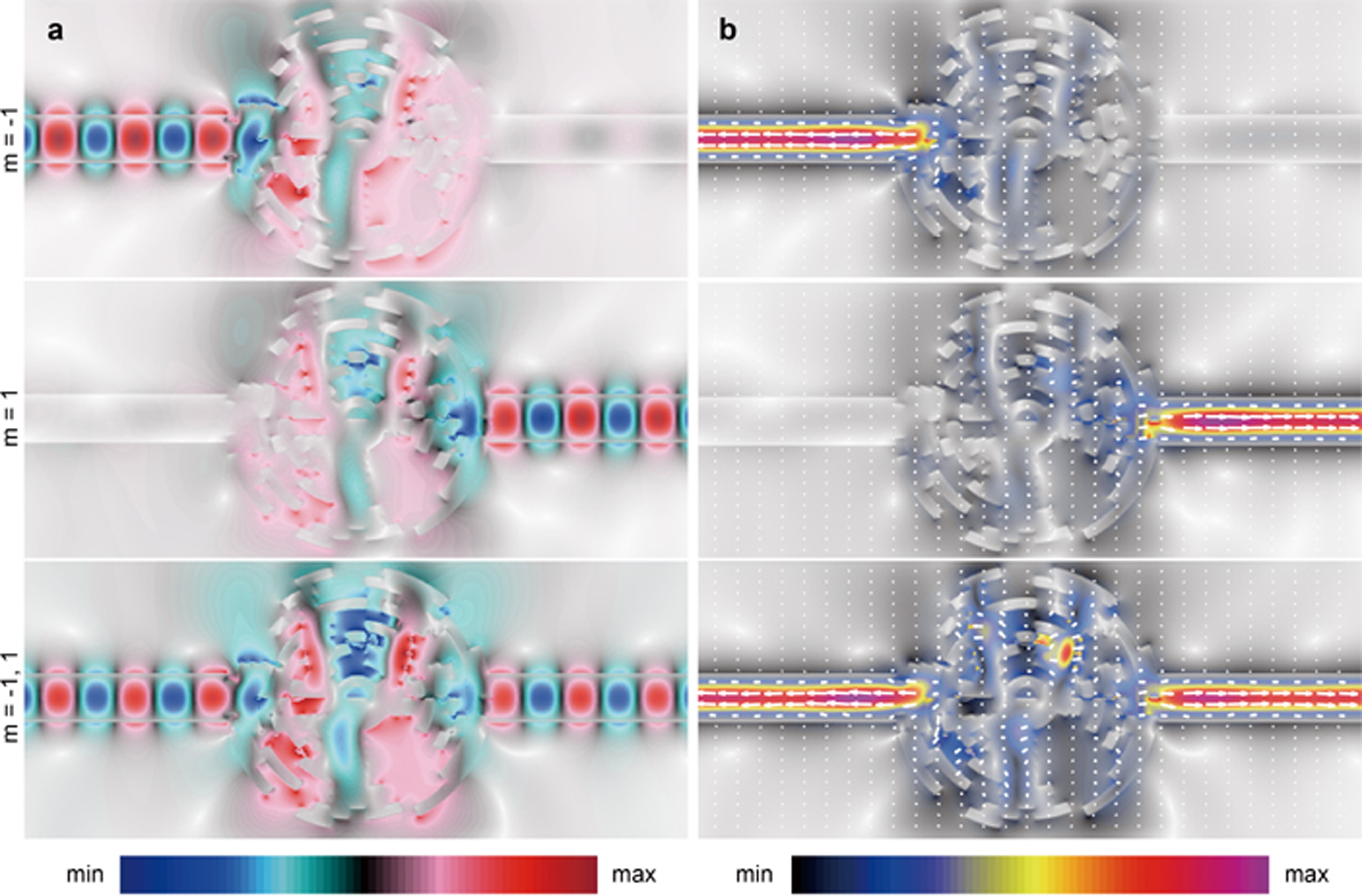

Supplement: Supplementary Figure S7 [file lsa20181x7.tif]

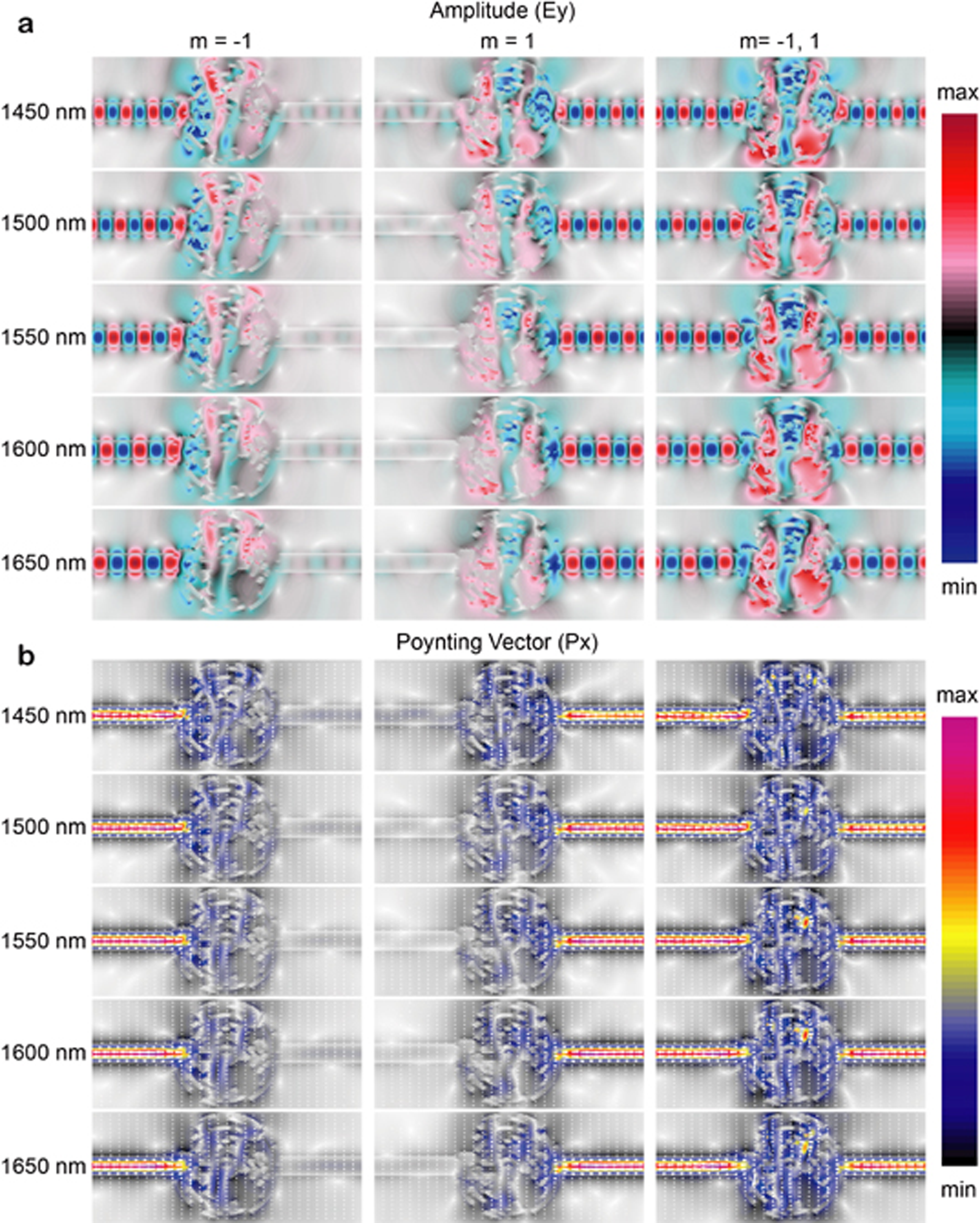

Supplement: Supplementary Figure S8 [file lsa20181x8.tif]

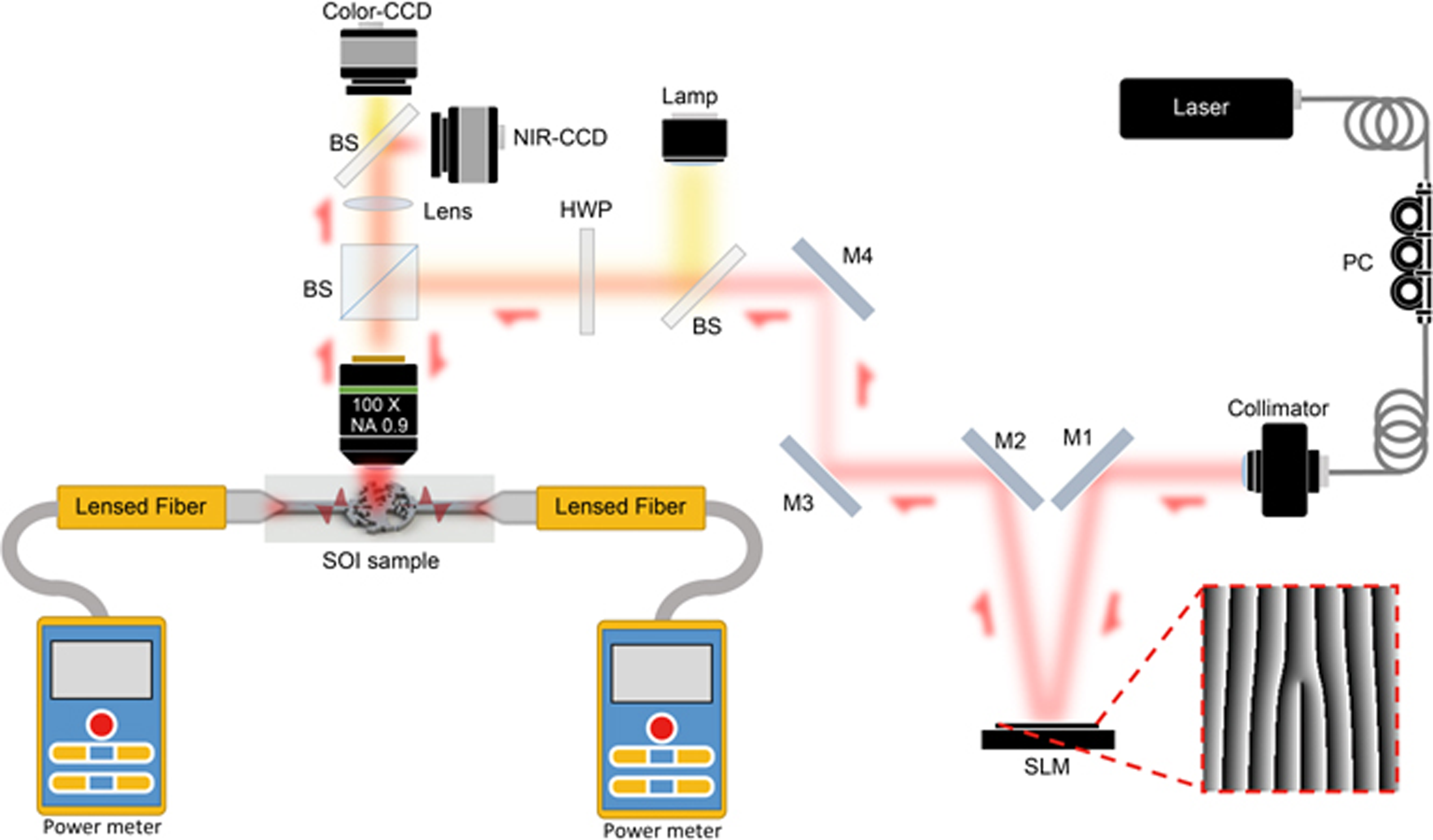

Supplement: Supplementary Figure S9 [file lsa20181x9.tif]

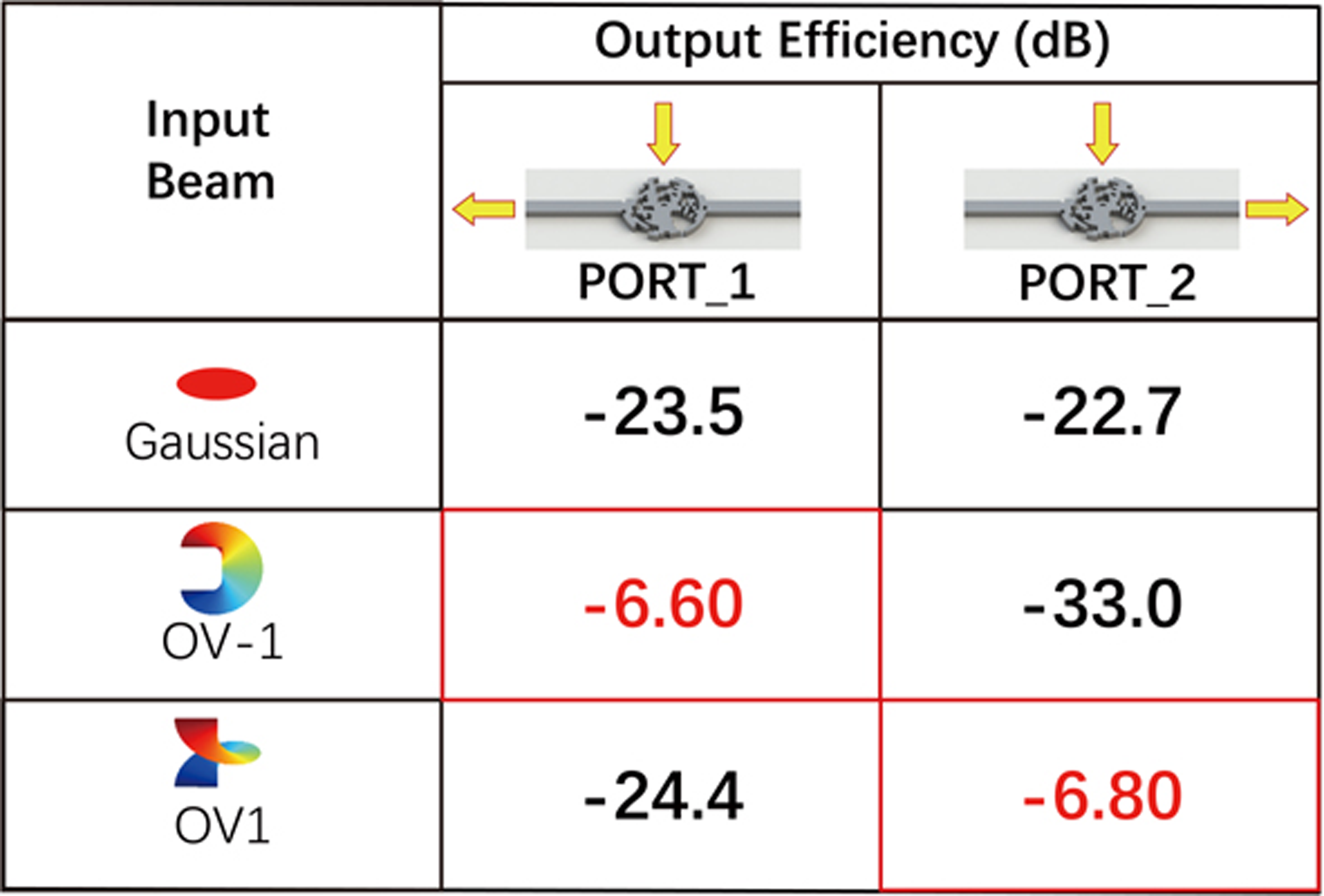

Supplement: Supplementary Figure S10 [file lsa20181x10.tif]

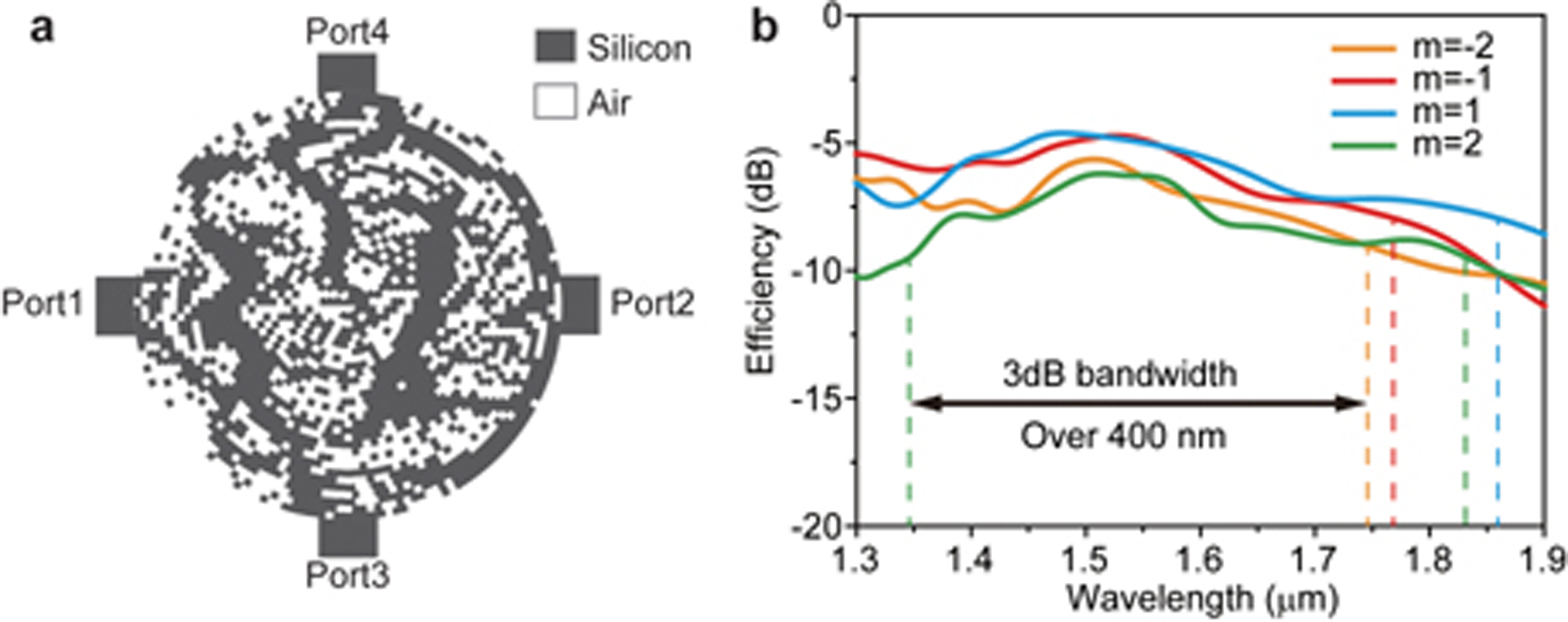

Supplement: Supplementary Figure S11 [file lsa20181x11.tif]

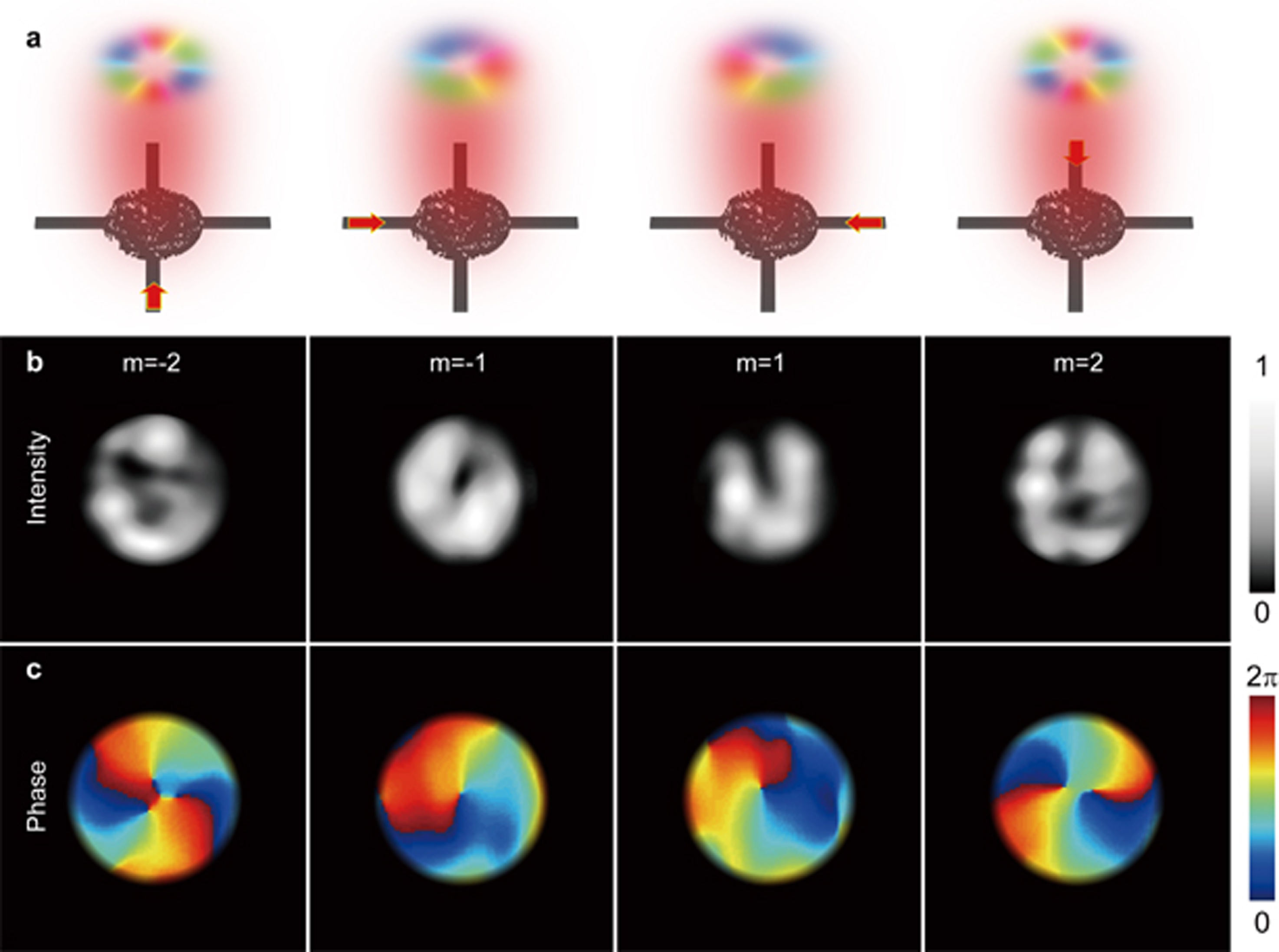

Supplement: Supplementary Figure S12 [file lsa20181x12.tif]

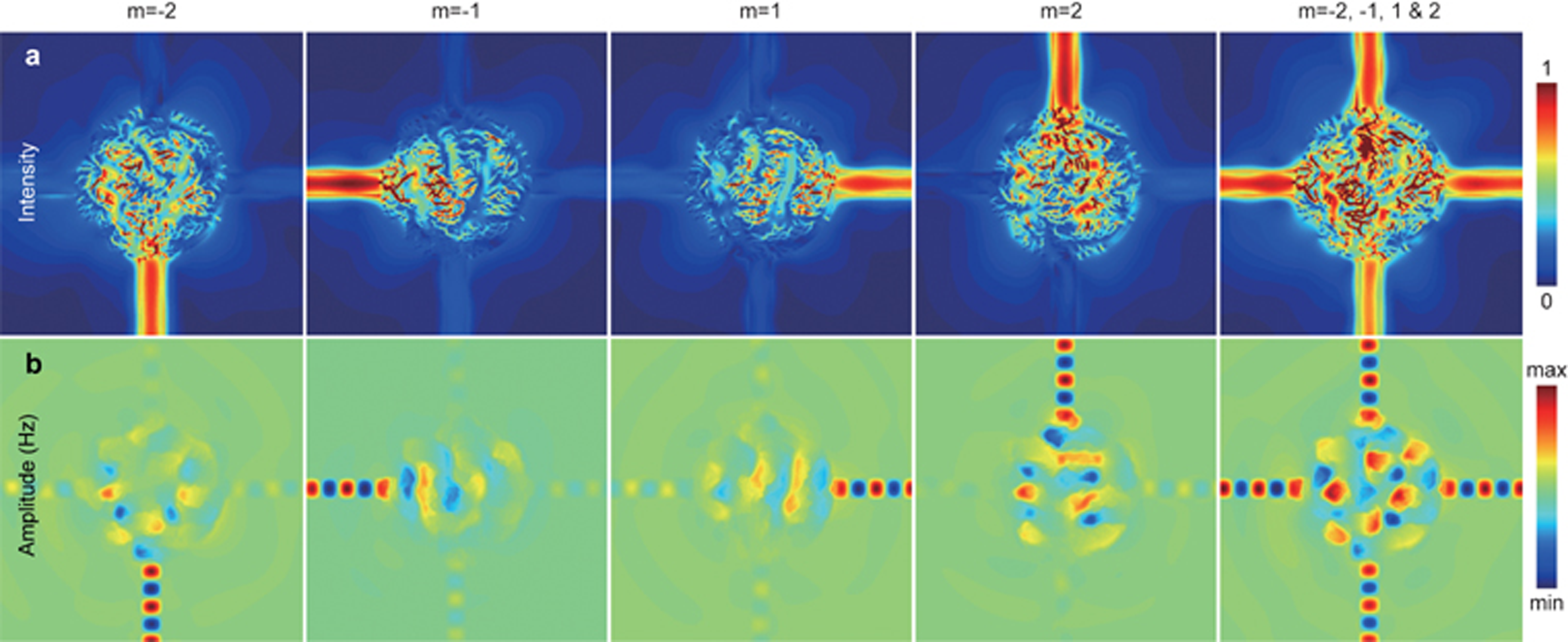

Supplement: Supplementary Figure S13 [file lsa20181x13.tif]

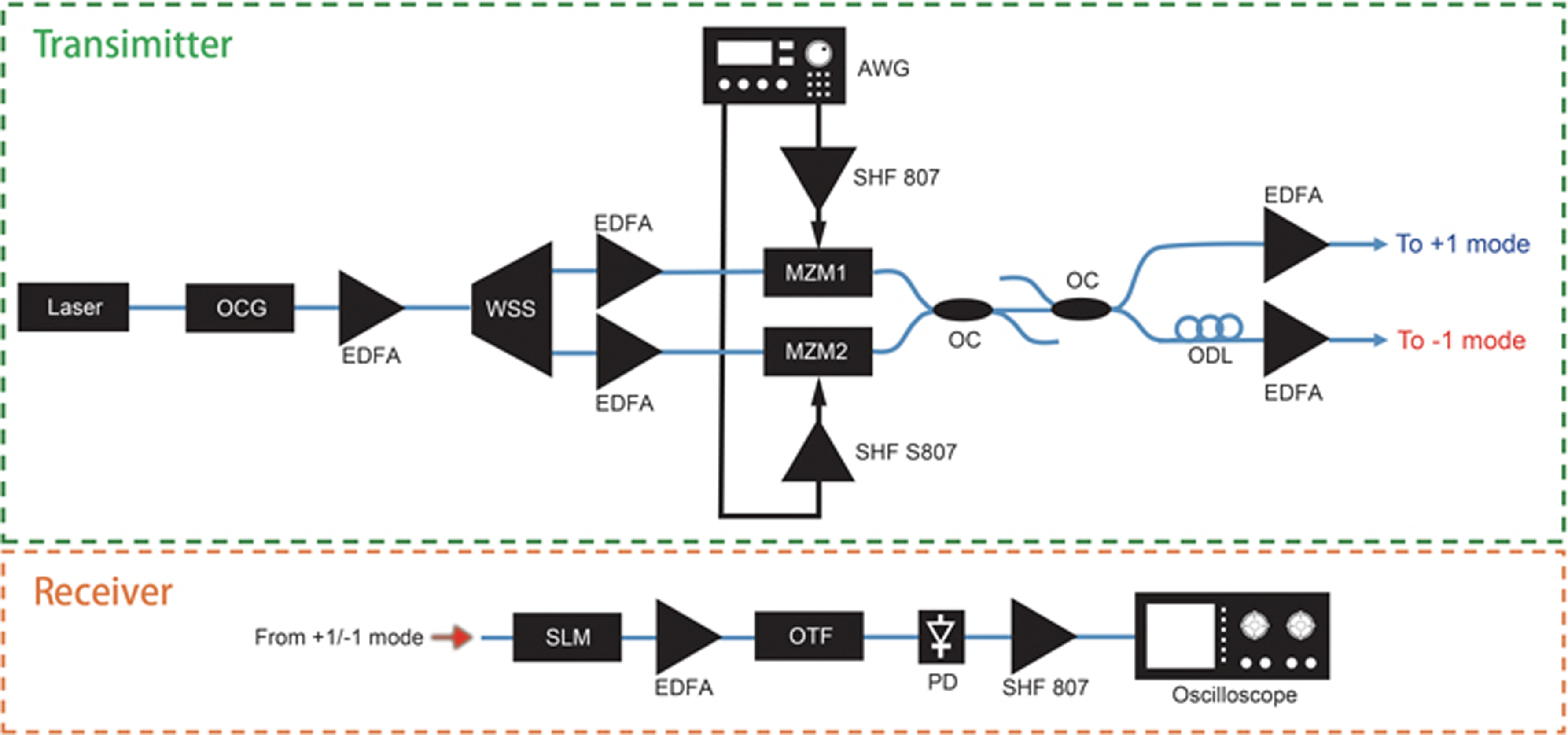

Supplement: Supplementary Figure S14 [file lsa20181x14.tif]

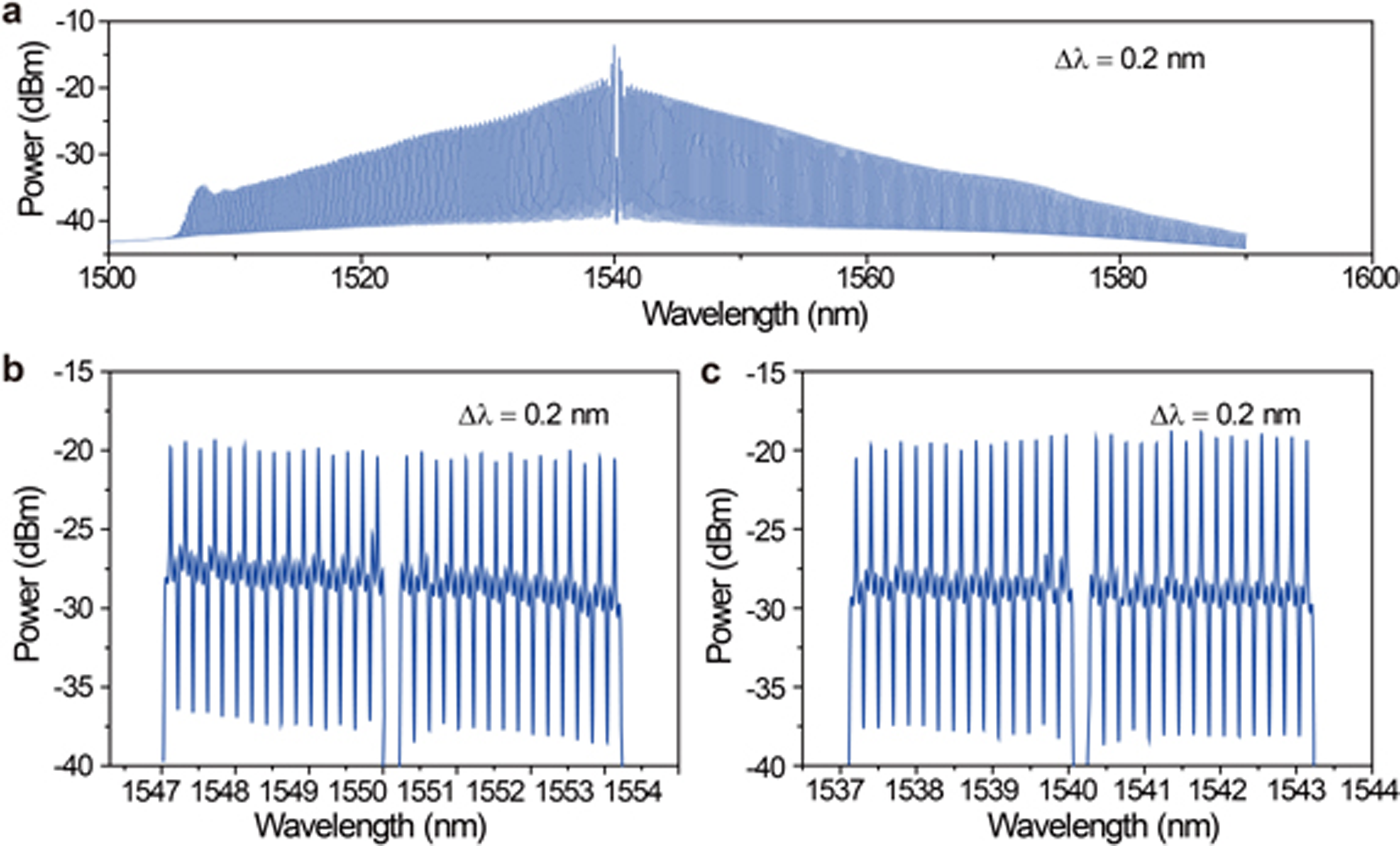

Supplement: Supplementary Figure S15 [file lsa20181x15.tif]
