# Supplementary material for: Ultra-broadband on-chip twisted light emitter for optical communications
Source: Light Sci Appl. 2018 Apr 20;7:18001–. doi: 10.1038/lsa.2018.1 (PMC6060059; doi:10.1038/lsa.2018.1)
Supplement: Supplementary Information [file lsa20181x16.doc]

Supplementary Information for

**Ultra-broadband On-chip Twisted Light Emitter for Optical Communications**

Zhenwei Xie^1†^, Ting Lei^1†*^, Fan Li^2^, Haodong Qiu^3^, Zecen Zhang^3^, Hong Wang^3^, Changjun Min^1^, Luping Du^1^, Zhaohui Li^2*^, Xiaocong Yuan^1*^

*^1^Nanophotonics Research Center, Shenzhen Key Laboratory of Micro-scale Optical Information Technology, Shenzhen University, Shenzhen, 518060, Guangdong, China.*

*^2^State Key Laboratory of Optoelectronic Materials and Technologies and School of Electronics and Information Technology, Sun Yat-sen University, Guangzhou 510275, China.*

*^3^ School of Electrical and Electronic Engineering, Nanyang Technological University, Singapore 639798.*

*Corresponding authors. E-mail: [xcyuan@szu.edu.cn](mailto:xcyuan@szu.edu.cn) (X. C. Yuan); [li_zhaohui@hotmail.com](mailto:li_zhaohui@hotmail.com) (Z. Li); [leiting@szu.edu.cn](mailto:leiting@szu.edu.cn) (T. Lei).

† Zhenwei Xie and Ting Lei contributed equally to this work.

**1. Phase delay of propagating and local resonance**

The total phase modulation during the OAM emission process originates both the propagation phase delay and the resonance phase delay from the local subwavelength structure. Thus the phase modulation for the emitter can be expressed as

(S1)

where are the polar coordinates (origin is the center of the device), is the phase modulation of the emitter, and are the phase modulations induced by the propagation and localized resonance, respectively. The propagation phase delay is calculated from the integral along the propagation path

(S2)

where $P(R,\pi)$ is the link point for the device and its left arm, , is the local point in the device where the light is emitted out of plane, is the refractive index distribution of the device determined by the detailed substructures. The propagation delay is linearly proportional to the propagation path of the light. The maximum optical path length for the proposed device is 2.4 um (the diameter of the device area), and the corresponding propagation delay can be calculated as

$\Delta t=\frac{nL}{c}=\frac{1.7\times2.4\times10^{-6}m}{3.0\times10^{8}m/s}\approx13.6\text{ fs},$ (S3)

where n is the estimated effective refraction index across the silicon and the air based pixels (The refraction index of the silicon is 3.48, and the refraction index of the air is 1. There are more air pixels than the silicon pixels in the optical path (about 5:2 ratio), so that the estimated effective refraction index is around 1.7), c is the speed of the light. The corresponding propagation phase delay is

(S4)

Thus, according to the Eqs. (S3) and (S4), the estimated propagation delay lies somewhere in the range of 0 to 6π, corresponding to a response time less than 15fs. The resonance phase delay is derived with respect to the local refractive index distribution and structure volume as follows:

$\phi_{2}(r,\varphi)=\frac{2\pi}{\lambda}N(r,\varphi)c\frac{Q}{\omega_{0}}+\phi',$ (S5)

where $\tau$ is the group delay caused by the local resonance, $\omega_{0}$ is the central frequency of the incident beam, $Q$ is the quality factor of the local cavity, is the difference between the phase delay and the group delay of the local resonance, and its value lies between -π~π. In a homogeneous media, when the group speed of the light is equal to the phase speed, there will be no difference between them, i. e. . For a more general situation, the value of the ranges from 0 to 2π. In order to indicate the advance and delay of the phase, we modify the value from 0~2π into -π~π. For a cavity with a volume of $V$, the quality factor can be expressed as

$Q=\frac{M\omega_{0}V}{D},$ (S6)

where *M* is the mode density of the cavity, $D$ is the mode degeneracy. For a small cavity, its mode degeneracy can be seen as 1, and the mode density can be written as

(S7)

Thus the quality factor of the cavity can be rewritten as

(S8)

We substitute the Eq. (S8) into the Eq. (S5); thus the is derived as

$\phi_{2}(r,\varphi)=\frac{8\pi N^{4}(r,\varphi)V}{D\lambda^{3}}+\phi'.$ (S9)

The cavity with the smallest volume would be the cavity which only contains one single pixel. Its volume can be calculated as

$V_{\min}=\frac{S_{\min}h}{\lambda^{3}}=\frac{100\text{nm}\times100\text{nm}\times220\text{nm}}{(1550\text{nm)}^{3}}\lambda^{3}\approx5.9\times10^{-4}\lambda^{3}$ (S10)

The cavity with the maximum volume can be obtained from the Figure S2b, which is about one sixth of the design area, and its volume can be calculated as

$V_{\max}=\frac{S_{area}h}{6\lambda^{3}}=\frac{\pi\cdot(1200\text{nm)}^{2}\times220\text{nm}}{6\cdot(1550\text{nm)}^{3}}\lambda^{3}\approx4.45\times10^{-2}\lambda^{3}$ (S11)

Considering that the substructures of the designed devices have volumes ranging from $5.9\times10^{-4}\lambda^{3}$ to $4.5\times10^{-2}\lambda^{3}$. The calculated phase delay is sufficient for a 2π phase modulation while the corresponding Q of the local cavity is below 15. Therefore, the designed device is equipped with fully phase control and ultrafast time response properties. According to the time-bandwidth product relation, the emitter should also have a broadband spectral domain. We substitute the Eq. (S2) and Eq. (S9) into the Eq. (S1); thus is derived as

(S12)

Considering that light coming from the left arm yields the *m*_1_ order OAM mode, and light coming from the right-hand-side yields the *m*_2_ order OAM mode, we obtain the following set of equations:

(S13)

**2. Numerical design and device fabrication**

2.1 Structure design


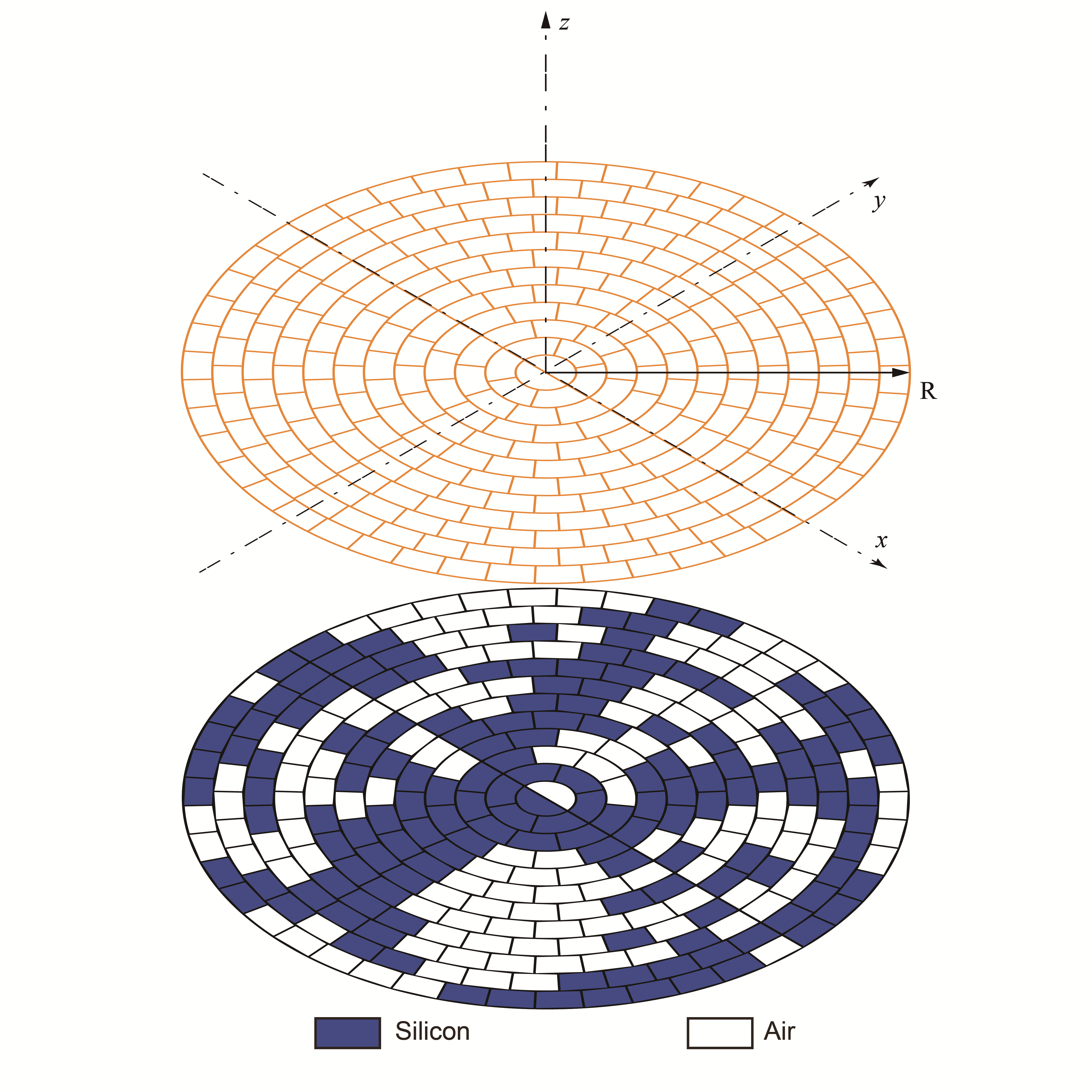


Figure S1 | Substructure design. The device area was circular with a radius of 1.2 μm, and the whole area was divided into 288 subpixels. The intervals in the radial direction were each 100 nm, while the azimuthal interval was also a little less than 100 nm. For each pixel the material was either silicon (blue) or air (white), with the material for each pixel being determined through an optimization algorithm associated with a 3D finite-difference time-domain (FDTD) simulation.

2.2 Fabrication process of the orbital angular momentum (OAM) emitter


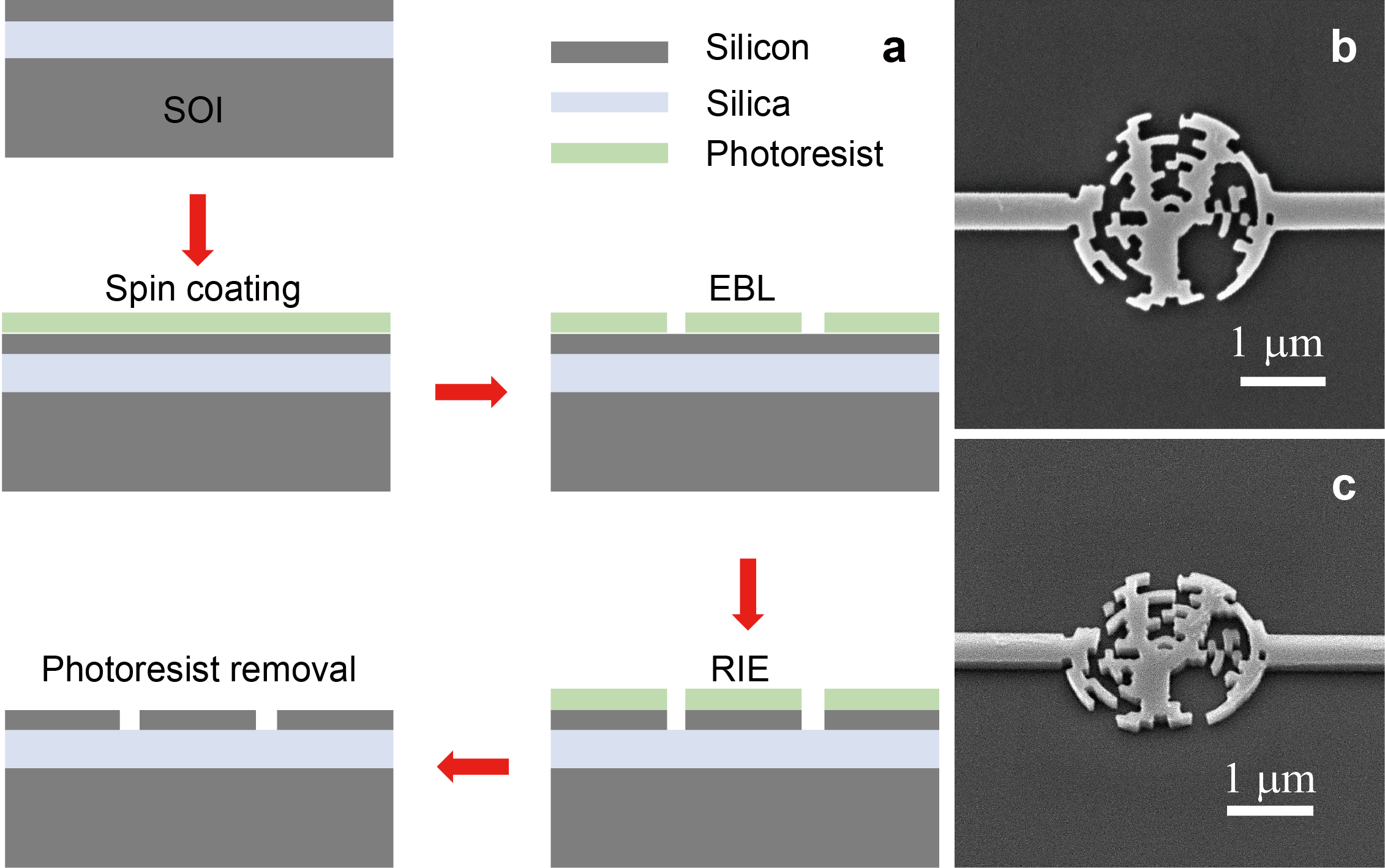


Figure S2 | Fabrication process and scanning electron microscope (SEM) images of the orbital angular momentum (OAM) emitter. a, The device was produced on a standard silicon on insulator wafer, with a 220 nm-thick device layer on top of a 2 μm-thick buried oxide layer. The process flow comprised photoresist spin coating, electron-beam lithography (EBL), deep reactive ion etching (RIE), and photoresist removal. b, c, SEM images of the device at two different perspectives: top view (b) and 45-degree view (c).

**3. Experimental methods**

3.1 Characterizing the intensity and phase profiles and emission efficiency

Fig. 3b in the main text shows the experimental setup for the characterization of the generated phase of the OAM modes and intensity profiles as well as for the emission bandwidth tests. The phase profiles were measured with a classical interferometer approach. After passing a polarization controller, the light from a laser that was tunable across the entire telecommunication band (from 1450 to 1650 nm, as shown in Fig. S4) was split into two branches by a 3 dB optical coupler. One branch was passed to a variable optical attenuator (2–60 dB) and a collimator to produce an expanded Gaussian beam as the reference beam. A half wave plate (HWP) was utilized to ensure a consistent polarization between the emission beam and the reference beam. The other branch was coupled into the input waveguide (left or right arm of the device) by a lensed fiber to generate the −1 or +1 OAM mode through the device. The generated OAM beam was collimated with an objective (100×, NA=0.9), and then split into two branches by a beam splitter. One branch of the OAM beam and the Gaussian reference beam were combined at a beam splitter, and then the interference patterns were captured with an infrared CCD camera (Hamamatsu InGaAs Camera C14041-10U, 320×256 pixels, pixel size 30 μm, high sensitivity in the near infrared region from 950 to 1700 nm, 216 frames/s). The other branch of the OAM beam was converted back to a quasi-Gaussian beam with a vortex hologram loaded onto a spatial light modulator (SLM); it was then coupled into a single mode optical fiber linked with an optical power meter to measure the output power. Here, a half wave plate was used to adjust the polarization of the output OAM mode to match that of the polarization demanded by the SLM. We used a white-light lamp as the illumination source, and a color CCD (Pointgrey Grasshopper GS3-U3-41C6C-C, 2048×2048 pixels, pixel size 5.5 μm, 90 frames/s) was utilized to observe and align the sample.


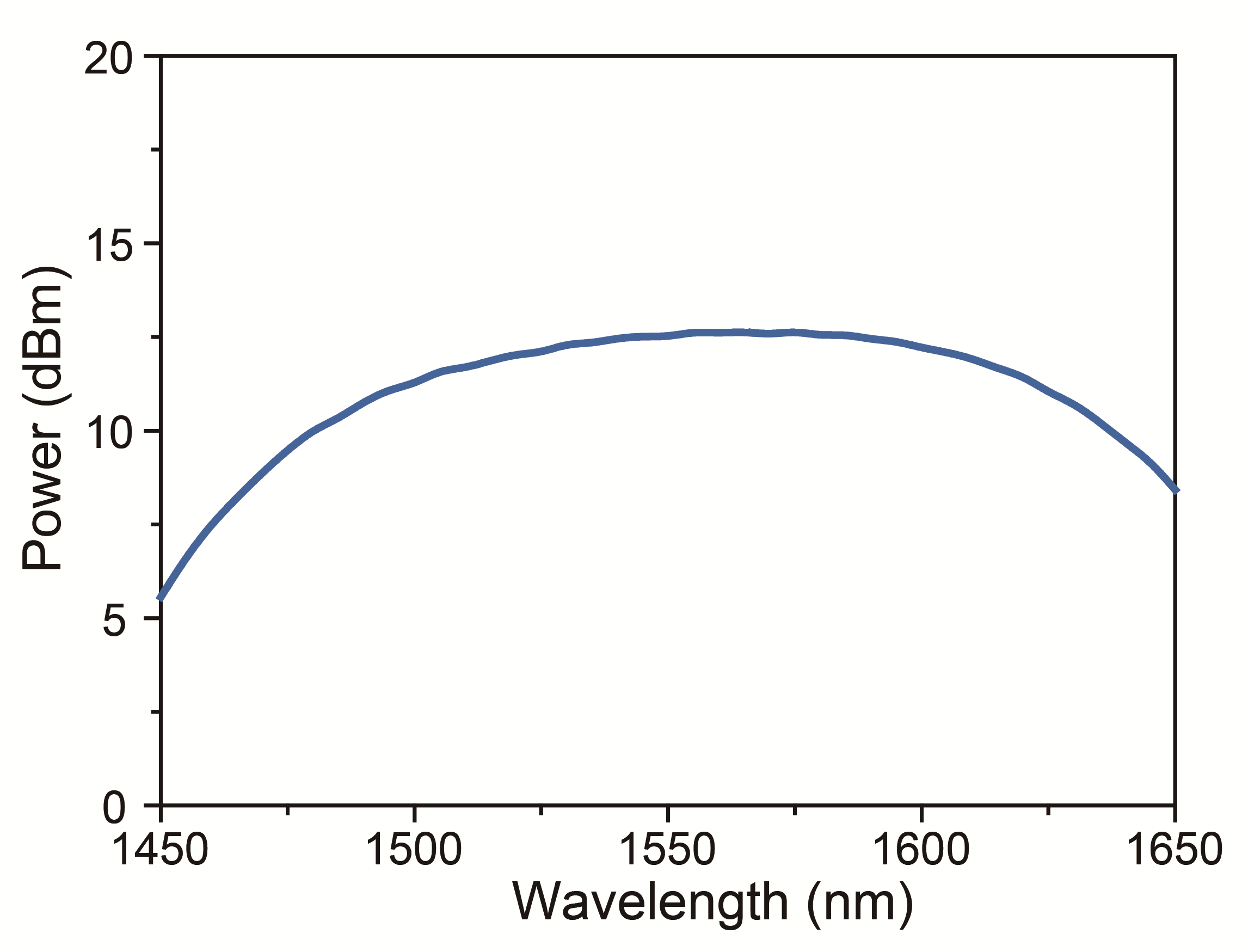


Figure S3 | The light source utilized to characterize the broadband spectrum of the device. It is a tunable laser (Keysight 81606A) with an output wavelength ranging from 1450 to 1650 nm.


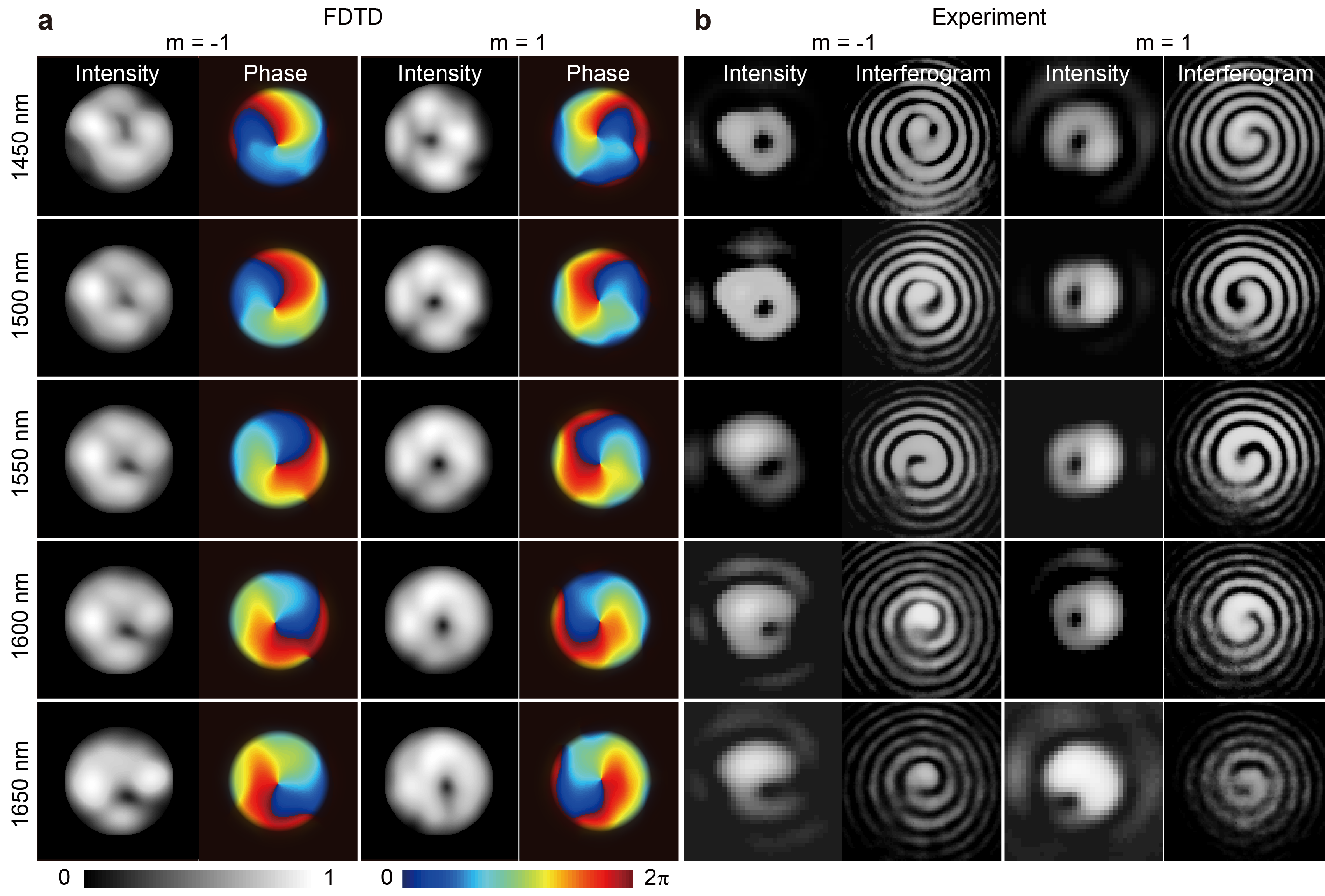


Figure S4 | FDTD simulation and Experimental measurements comparison for the intensity and phase profiles of the generated OAM modes. a, FDTD simulation for far-field intensity and phase distributions for the emission of the −1 and 1 OAM modes at wavelengths of 1450, 1500, 1550, 1600, and 1650 nm. b, Measured far-field intensity distribution and interference patterns for the emission of the −1 and 1 OAM modes at wavelengths of 1450, 1500, 1550, 1600, and 1650 nm.


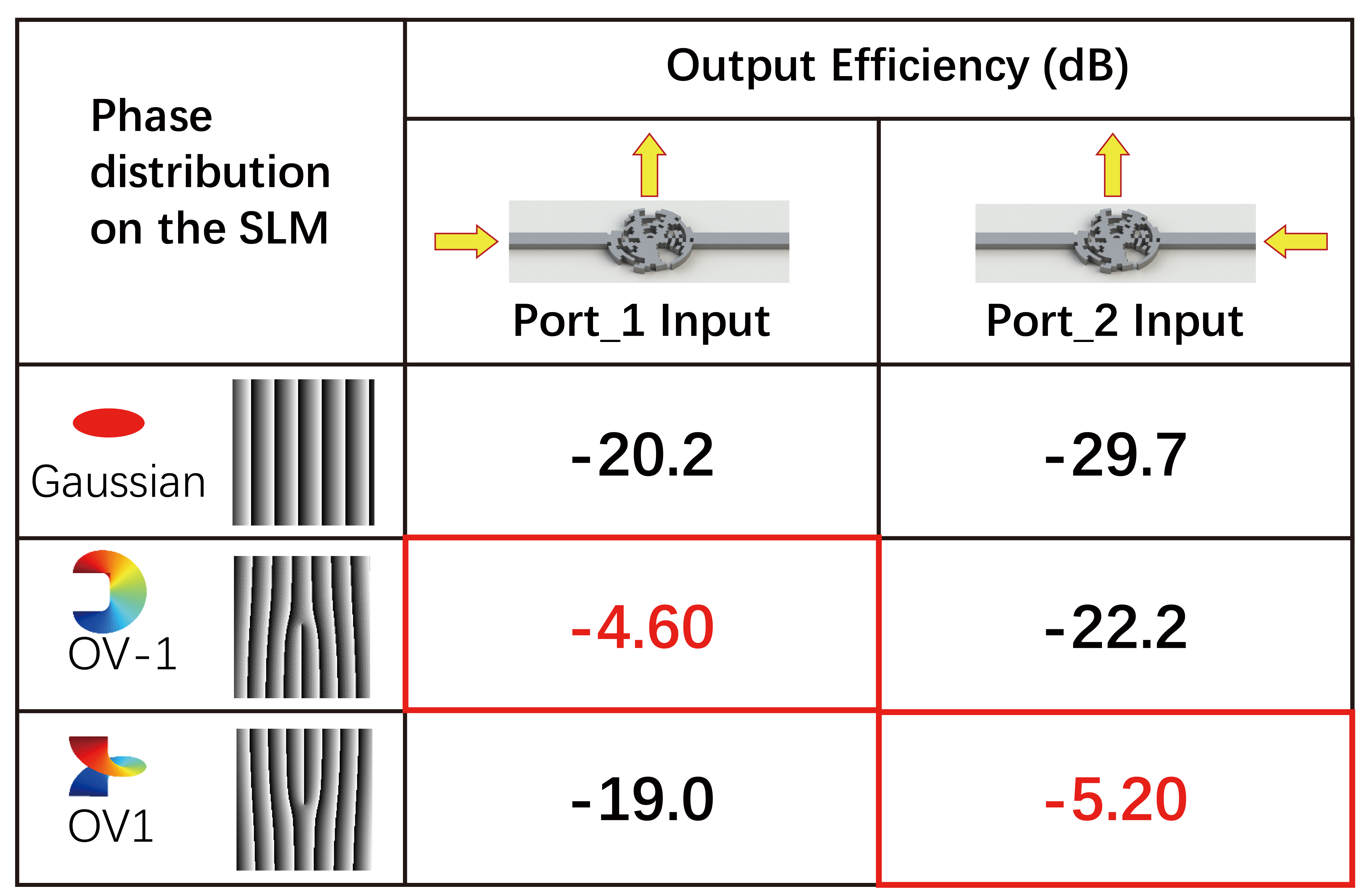


Figure S5 | Emission efficiency and mode purity measurement at 1550 nm. The generated OAM was tested using a vortex hologram loaded in the SLM and then coupled into a single mode optical fiber. The output power was measured with an optical power meter and the corresponding efficiency was calculated by taking into account of the insertion loss of SLM, objective lens, beam splitter, and the coupling loss of the lensed fiber.

The intensity distribution was measured by blocking the reference branch in the interferometer. The measured results at 1450, 1500, 1550, 1600, and 1650 nm are shown in Fig. 3 in the main text. The full comparison between the FDTD simulation and the experimental measurements are shown in Fig. S4.

3.2 OAM demultiplexing and device alignment

The proposed OAM emitter could also be utilized as an OAM demultiplexer. Firstly, FDTD simulation was performed to verify the validity of the device, and the results at 1550nm and other wavelength are illustrated in Figs S6 and S7. The experimental set-up for the OAM demultiplexing and the alignment between the device and the optical axis of the measurement system is illustrated in Fig. S8. The set-up was similar to the OAM emission system but with a reverse process. The light from a tunable laser passed through a polarization controller and was then collimated into a Gaussian beam by a collimator. After modulation by a vortex hologram loaded onto a SLM, the Gaussian beam was converted into an OAM beam, and its topological charge was determined by the hologram. The OAM beam was focused onto the device by an objective (100× NA=0.9), and its polarization was adjusted with a HWP to match the operation polarization of the device. The output power from both arms was measured through lensed fibers with optical power meters. A white light lamp was utilized as the illumination source and a color CCD was used for the observations.


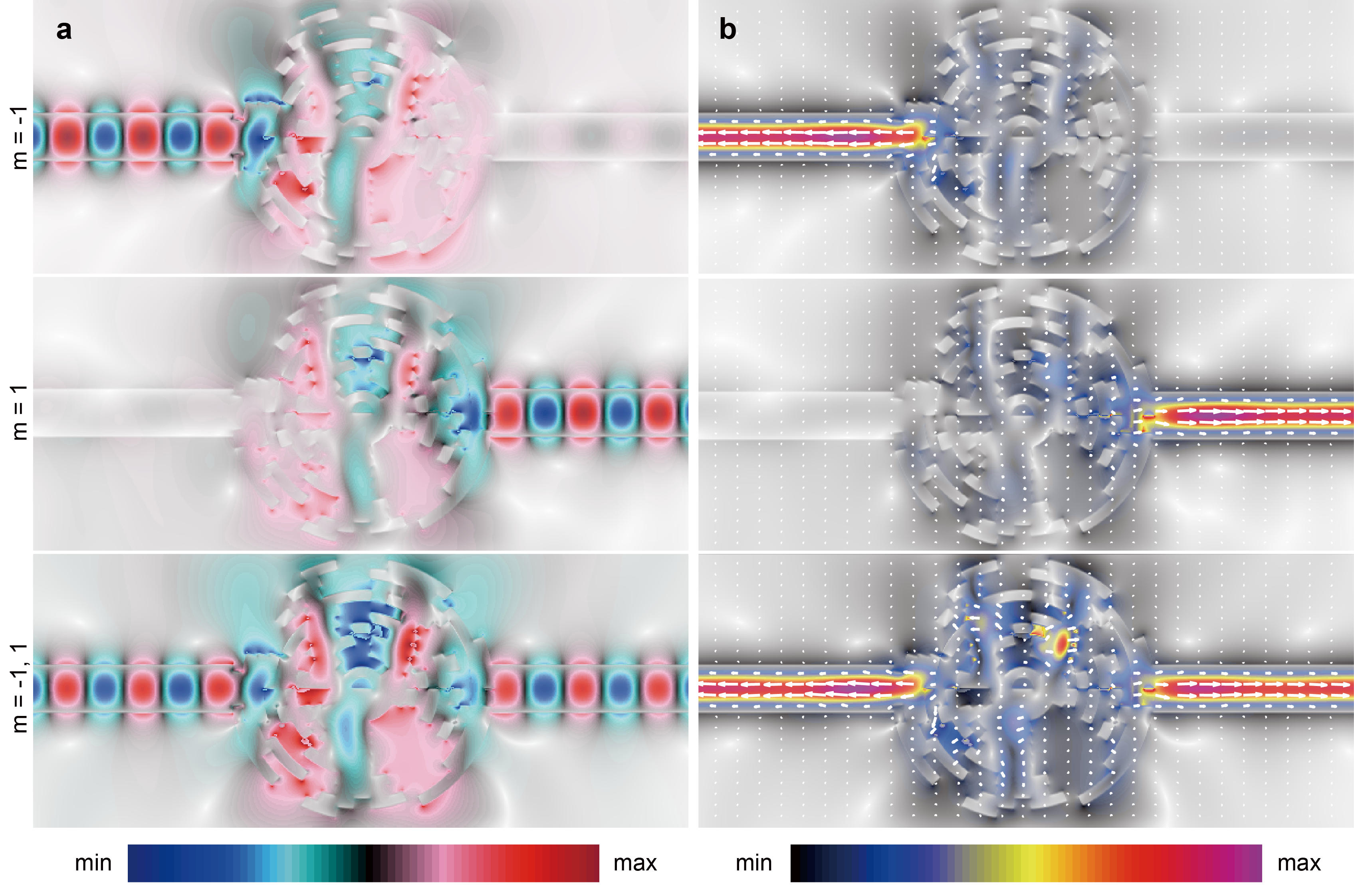


Figure S6 | FDTD simulation for the device as an OAM demultiplexer at 1550 nm for an input beam of OAM m=+1, OAM m=−1, or OAM m=±1. The device couples the −1 mode to its left arm and the +1 mode to its right arm. a, The amplitude distributions for the *Ey* components. b, The profile for the Poynting vector, where the white arrows indicate the direction of the energy flow.


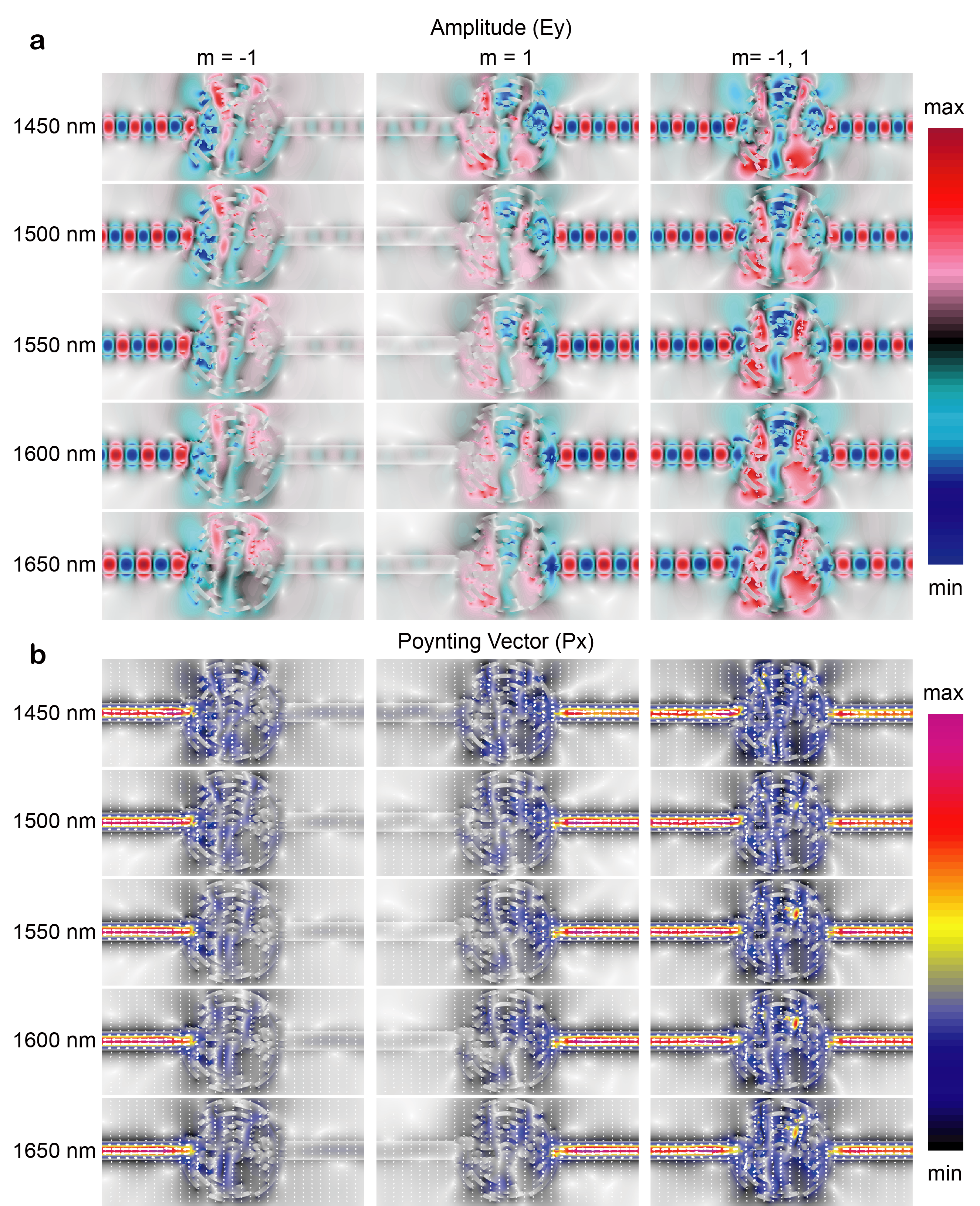


Figure S7 | FDTD simulations for the device as an OAM demultiplexer at 1450, 1500, 1550, 1600, and 1650 nm for an input beam of OAM m=+1, OAM m=−1, or OAMs m=±1. The device works well over the 200 nm-wide regime. a, The amplitude distributions for the *Ey* components. b, The profile for the Poynting vector, where the white arrows indicate the direction of the energy flow.


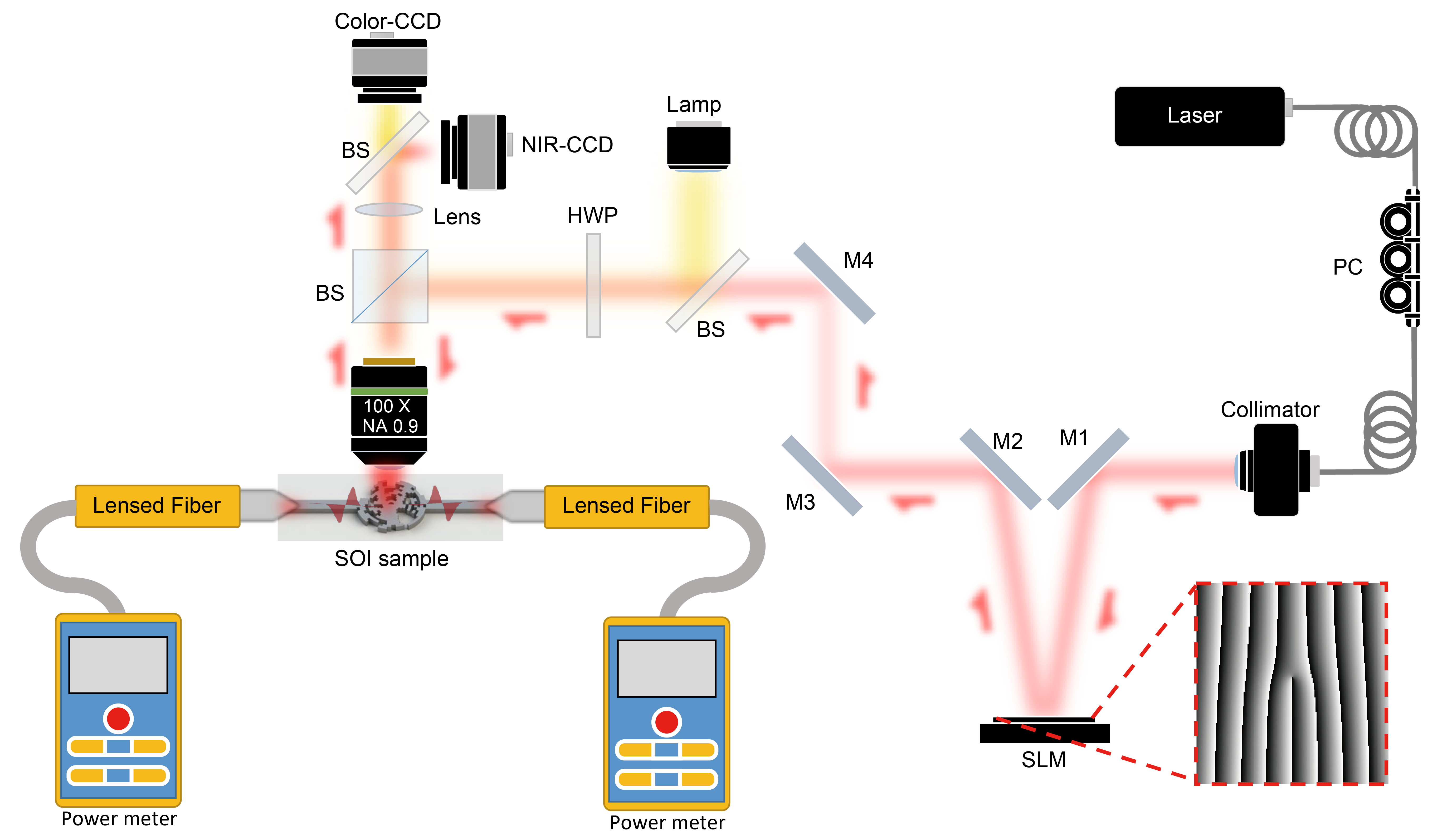


Figure S8 | The experimental set-up for OAM demultiplexing. This set-up was also utilized for the alignment between the device and the optical axis of the measurement system. The light from a tunable laser passed through a polarization controller and was then collimated into a Gaussian beam by a collimator. After being modulated by a SLM loaded with a vortex hologram, the Gaussian beam was converted into an OAM beam, and its topological charge was determined by the hologram loaded on the SLM. The OAM beam was focus onto the device by an objective (100× NA=0.9), and its polarization could be adjusted with a HWP to match the operation polarization of the device. The output power from both arms was measured with optical power meters linked to lensed fiber used to couple the light from the waveguides. A white light lamp was utilized as the illumination source, and a color CCD was used for the observations.


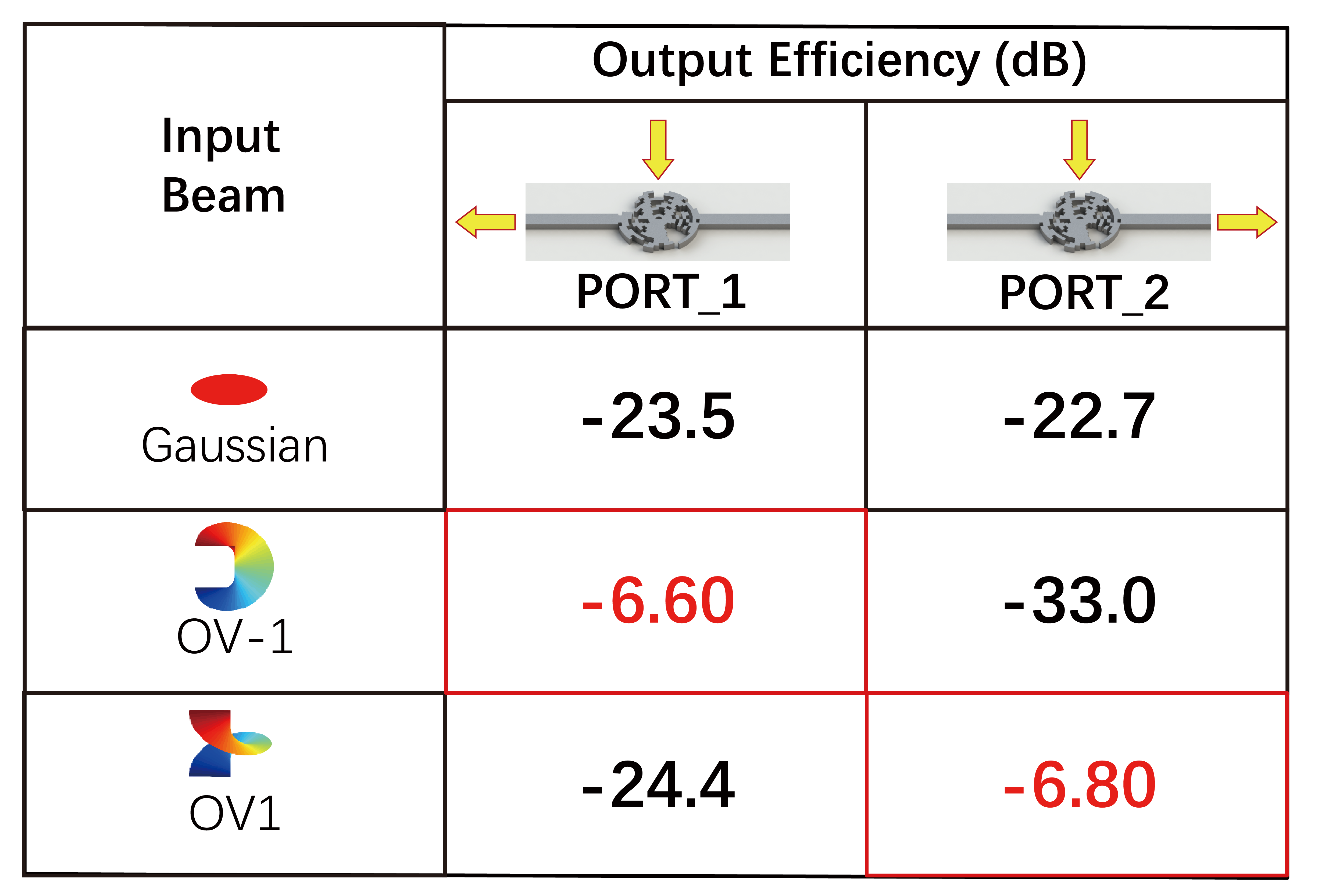


Figure S9 | The efficiency for OAM detection and for the channel segregation measurement for two OAM modes at 1550 nm. The OAM beam is generated with a vortex hologram loaded onto a SLM and then illuminated onto the device. The light was coupled into either the left or right arm of the device depending on the topological charge of the incident OAM beam. The output power was measured with an optical power meter and the corresponding efficiency was calculated by taking into account of the insertion loss of SLM, objective lens, beam splitter, and the coupling loss of the lensed fiber.

3.3 Design and simulations for four ports OAM emitter

In order to verify that the propose approach could be adapted to a device design for simultaneously emitting a large number of OAM modes, we designed and demonstrated a four ports OAM emitter with FDTD simulations. The material is still standard Silicon on insulator with a 220 nm thick device layer on a 2 μm thick buried oxide layer. Here the design area is set as a circular with a radius of 1.5 μm. Then the area is divided into 2670 pixels with a feature size of 50 nm. Considering that light coming from the left arm yields the *m*_1_ order OAM mode, light coming from the right-hand-side yields the *m*_2_ order OAM mode, light coming from the down-side yields the *m*_3_ order OAM mode, light coming from the up-side yields the *m*_4_ order OAM mode, we obtain the following set of equations:

(S14)

Then by using the proposed global optimization algorithm to solve the equation set , we can obtain that the specific structure design of the device. Here we choose the *m*_1_, *m*_2_, *m*_3_ and *m*_4_ as -1, 1, -2 and 2, respectively. The specific structure design is shown in Fig. S10a. In order to verify its working bandwidth, we investigated the mode emitting efficiency for all the four OAM modes at wavelength from 1.3 μm to 1.9 μm. From the simulation results presented in Fig. S10b, we can see that the over all 3 dB bandwidth of the device is over 400 nm. The maximum emitting efficiency can reach to 37 % at wavelength of 1500 nm. We also investigated the far field profiles of the generated OAM modes. The results are shown in Fig. S11. The Fig. S11a shows the schematics for procedures that how the light coming from each port of the device is converted into a free space OAM mode. The far-field intensity profiles for all four OAM modes at wavelength of 1550 nm are illustrated in Fig. S11b. We can see that there are some inhomogenous distributions in the intensity profiles. The main reason is that the emitting efficiency for each small pixel or structure of the device is not the same. This effect can be subdued by adding some extra factor in the global optimization. While the corresponding phase profiles are shown in Fig. S11c, and we can clearly see the phase change around the center are -4π, -2π, 2π and 4π, respectively. This indicates that the corresponding topological charges of the OAM modes are -2, -1, 1 and 2, respectively.

It is obvious that the device can also be used as an OAM modes demultiplexer. The corresponding numerical simulations are shown in Fig. S12. The intensity profiles at the device plane in Fig. S12a show the situations when the incident beam are OAM mode m=-2, m=-1, m=1, m=2 and OAMs m=-2, -1, 1 and 2, respectively. From the amplitude of the Hz component in Fig. S12b, we can see how the light propagating more clearly. For incident OAM modes with topological charge of -2, -1, 1 and 2, they will be coupled into the waveguides of down-side, left-side, right-side and up-side, respectively. Here the waveguides are all single mode ones which only support the fundamental TE mode. Therefore, in order to investigate the light propagating in the waveguide, we have to monitor the common component of waveguide spreading to different directions. That is why we choose to monitor the Hz component here.

Here we want to state that the proposed approach is actually universal to the design for all kinds of different the OAM modes with different topological charge. Although we numerical demonstrated with topological charges of -2, -1, 1 and 2, there actually no limit in choosing OAM modes, and one may change it accordingly.


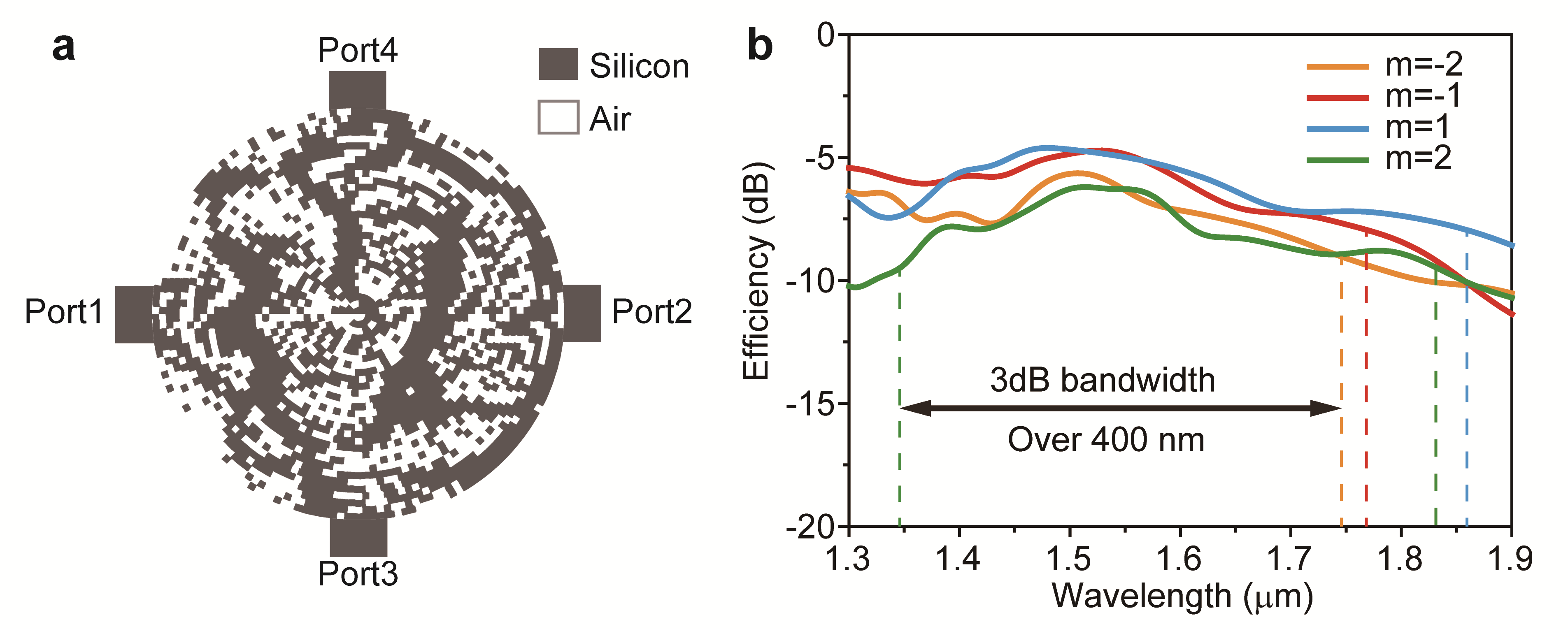


**Figure S10 | The four ports device for four OAM modes simultaneously emission/detection.** **a**, The design structure of the circular shaped device with a radius of 1.5 μm and pixels feature size of 50nm. **b**, The FDTD simulation of the bandwidth for the four OAM modes generated by the emitter. The maximum emitting efficiency can achieve 37%. The 3dB bandwidth of the OAM modes emitting is over 400 nm.


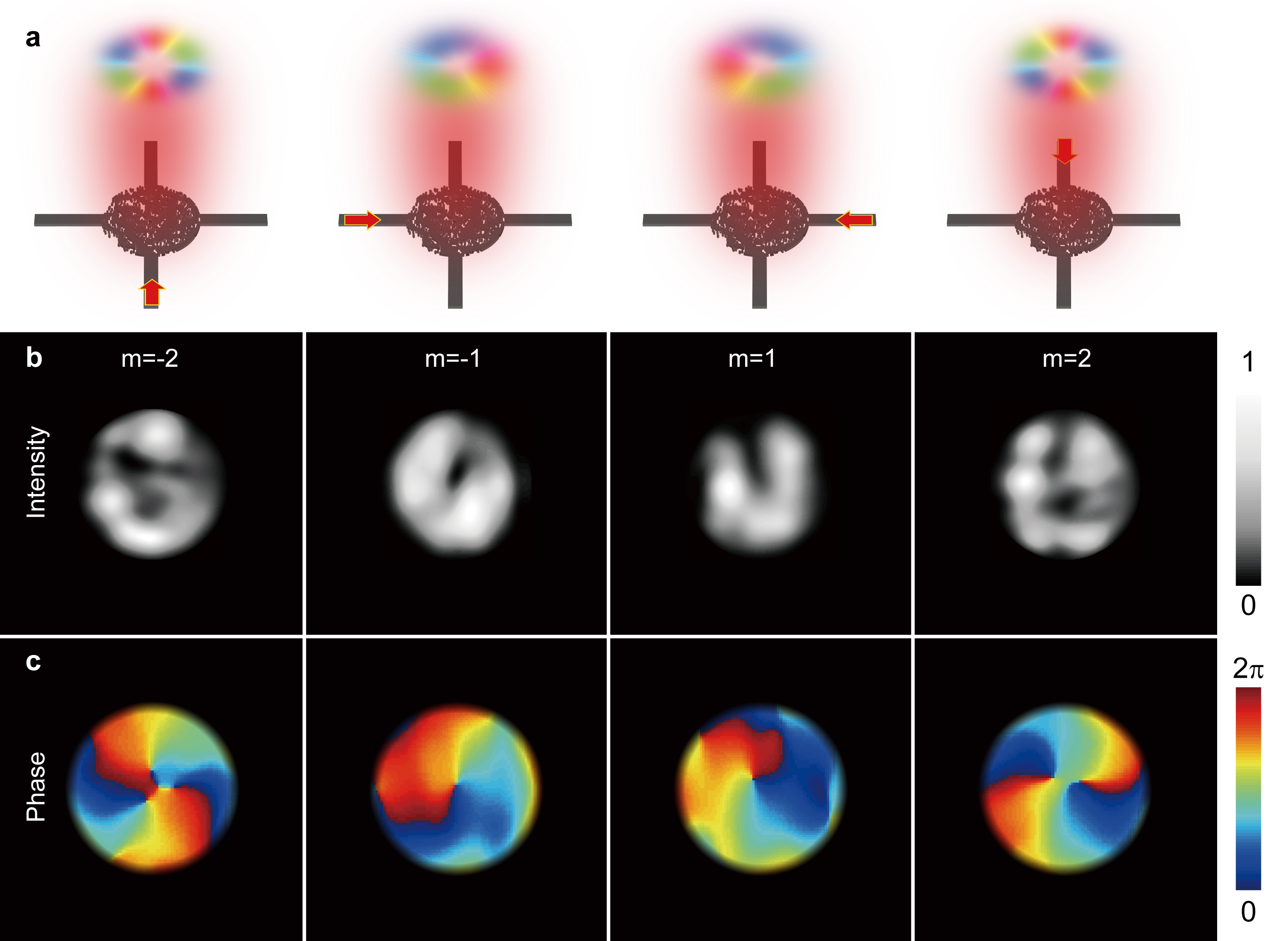


Figure S11 | FDTD simulations of the emitter generating four OAM modes with topological charge of m=-2, -1, 1 and 2, respectively. a, Schematics of the OAM emitter converting light from different ports to different OAM modes. b, The far-field intensity profiles of different generated OAM modes. c, The corresponding phase profiles of the OAM modes.


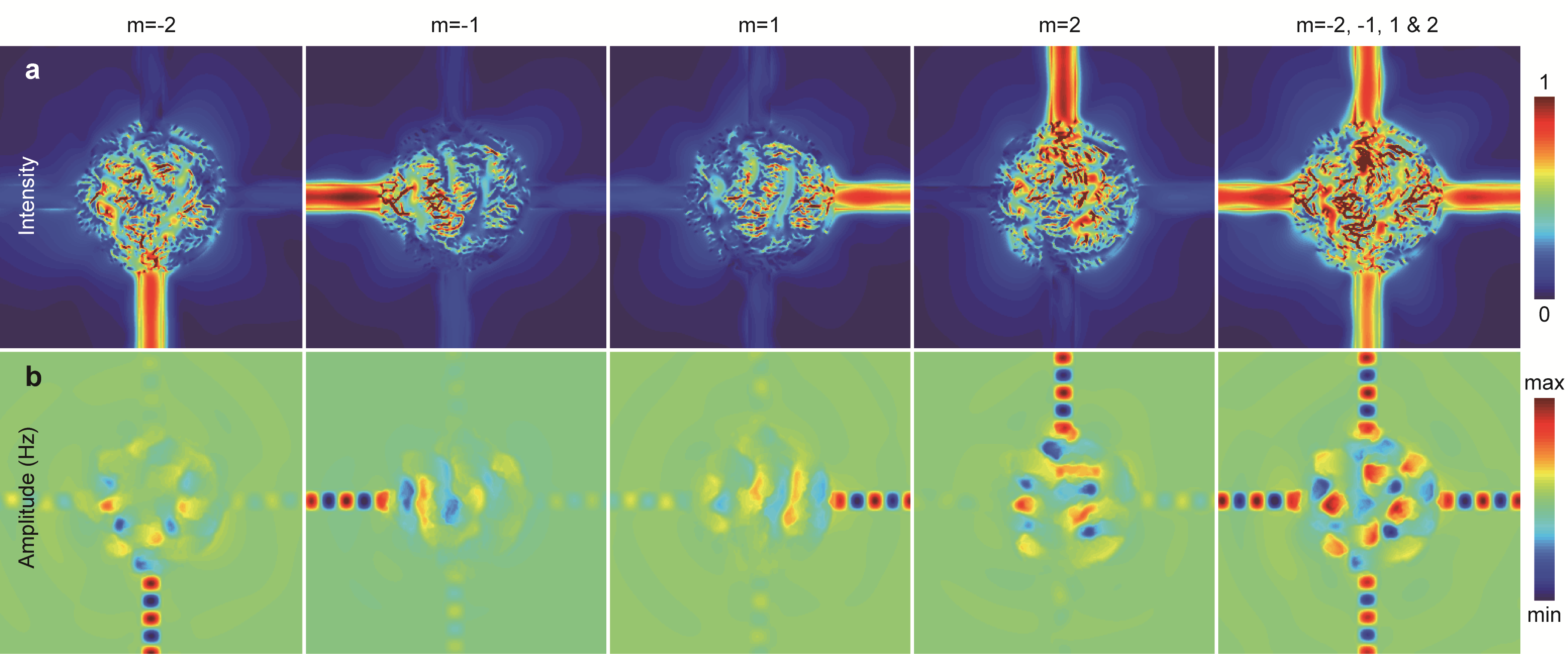


Figure S12 | FDTD simulations of the OAM demultiplexer for an input beam of OAM m=-2, OAM m=−1, OAM m=1, OAM m=2, and four OAMs m=±2 & ±1. a, The intensity distributions of the *E* components in the device plane. b, The amplitude profile for the *Hz* components in the device plane.

3.4 FDM and OAM (de)multiplexing communication

A schematic for quadrature phase shift keying (QPSK) and quadrature amplitude modulation (32QAM) optical communication is shown in Figure S13. The scheme comprises a transmitter system, an OAM emission/sorting and transmission system, and a receiver system. The set-ups for the OAM emission/sorting and transmission systems are shown in Figs. 3b in the main text and Fig. S8, respectively; only the transmitter and receiver parts are illustrated.


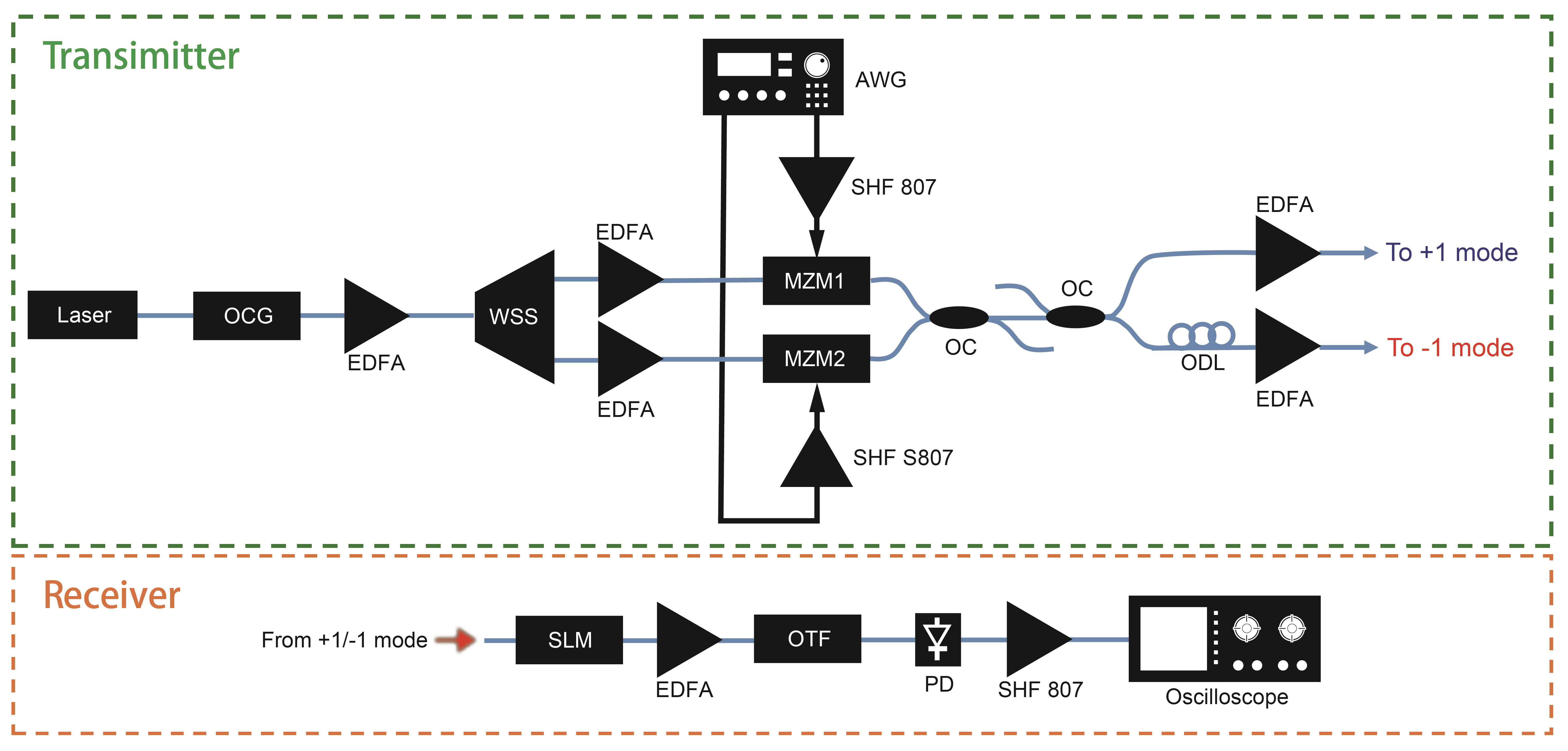


Figure S13 | Schematic for quadrature phase shift keying (QPSK) and quadrature amplitude modulation (32QAM) optical communication. For the transmitter, a tunable laser (Agilent N7711A, C-band 100 kHz linewidth) was utilized as the pump source. The generated frequency division multiplexing (FDM) subcarriers with a frequency separation of 25 GHz from an optical comb generator (WTAS-02) were amplified by an erbium-doped fiber amplifier (EDFA); the highly nonlinear fiber broadened the original frequency comb. A programmable optical wavelength selective switch (Finisar WaveShaper 4000S) equalizes the power of all the optical subcarriers (30-waves), and these subcarriers were then separated into even and odd subcarriers. Both sets of subcarriers were then individually modulated with independent QPSK/32QAM pseudorandom bit sequences. The FDM signal was subsequently generated by combining the even and odd subcarriers. Here the modulation signals were generated with an arbitrary-waveform-generator (Tektronix AWG70002A, sampling rate 25GS/s) and then amplified with a linear amplifier (SHF 807 with a bandwidth of 30 GHz or SHF S807 with a bandwidth of 55 GHz). These electric signals were transformed into optical signals using a Mach-Zehnder electro-optical modulator (MZM). The generated FDM signals were separated into two branches by an optical coupler. One branch was amplified with an EDFA and was then directly coupled to the right arm of the device by a lensed fiber to generate the FDM +1 OAM mode. The other branch was delayed with an optical delay line (ODL) with a 1 ns delay to ensure the signals of the two OAM modes were different; then the signal was amplified and coupled to the left arm of the device. The receiver comprised a spatial light modulator (SLM), an optical tunable filter (OTF, Santec OTF-350), a photodiode (PD, U2T XPDV2120R), a linear amplifier (SHF S807, bandwidth 50 GHz), and a real-time oscilloscope with a 50 GSa/s sampling rate (Tektronix DSA72004B).


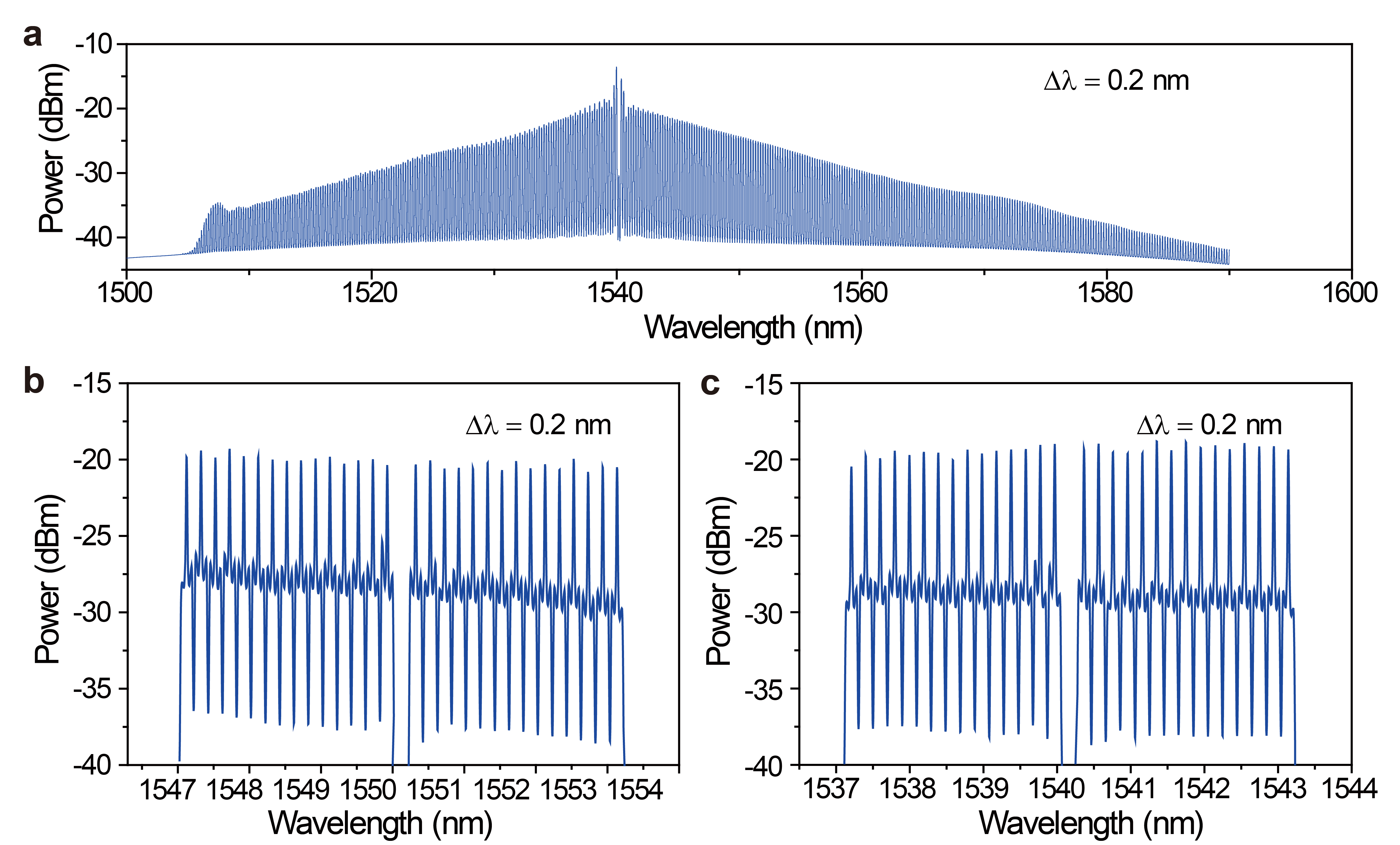


Figure S14 | Frequency comb for OFDM communication measurements. a, Femtosecond optical frequency comb from an optical frequency comb generator. b, The 30-wave FDM signal (from 1547 to 1554 nm, wavelength interval of 0.2 nm) for OAM mode multiplexing communication (here the emitter was used as a transmitter). These 30-wave channels were filtered out from the femtosecond optical frequency comb with a wave shaper. c, The 30-wave FDM signal (from 1537 to 1544 nm, wavelength interval of 0.2 nm) for OAM mode demultiplexing communication (the emitter was used as a receiver).

In the transmitter, a tunable laser (Agilent N7711A, C-band 100 kHz linewidth) was utilized as the pump source. The FDM subcarriers with a frequency separation of 25 GHz (were generated by an optical comb generator (WTAS-02)) were amplified by an erbium-doped fiber amplifier (EDFA); the highly nonlinear fiber broadened the original frequency comb. A programmable optical wavelength selective switch (Finisar WaveShaper 4000S) equalized the power of all 30 optical subcarriers (30-waves), and these subcarriers are then separated into even and odd subcarriers. Both sets of subcarriers were then individually modulated with independent QPSK/32QAM pseudorandom bit sequences. The FDM signal was generated by combining the even and odd subcarriers subsequently. Here the modulation signals were generated with an arbitrary-waveform-generator (Tektronix AWG70002A, sampling rate 25 GS/s), and then amplified with a linear amplifier (SHF 807 with a bandwidth of 30 GHz or SHF S807 with a bandwidth of 55 GHz). These electric signals were transformed to optical signals by a Mach-Zehnder electro-optical modulator. The generated FDM signals were separated into two branches by an optical coupler. One branch was amplified with an EDFA and then directly coupled to the right arm of the device via a lensed fiber to generate the FDM +1 OAM mode. The other branch was delayed with an optical delay line with a 1 ns delay to ensure the signals of the two OAM modes were different; then, the signal was amplified and coupled to the left arm of the device. For the OAM demultiplexing, the two branches of the FDM signals were sent to a SLM to generate the different coaxial OAMs, and the device was utilized to sort these modes and then couple the signals to each arm according to the topological charge of the OAM.

The receiver consisted of a SLM, an EDFA, an optical tunable filter (OTF, Santec OTF-350), a photodiode (PD, U2T XPDV2120R), a linear amplifier (SHF S807, bandwidth 50 GHz), and a real-time oscilloscope with a 50 GSa/s sampling rate (Tektronix DSA72004B). Here, the SLM was utilized for converting the OAM mode beams back to quasi-Gaussian beams and then couple them to a single mode optical fiber. The received signals were amplified with the EDFA, and then each subcarrier could be extracted with the tunable narrow bandpass filter (Santec OTF-350). The light signals were transformed into electrical signals by the photodiode. After amplification with a low noise amplifier, the subcarrier signals were captured with a real time oscilloscope. For the OAM demultiplexing, the SLM was utilized as the OAM mode emitter; thus, the proposed device functions as an OAM sorter.


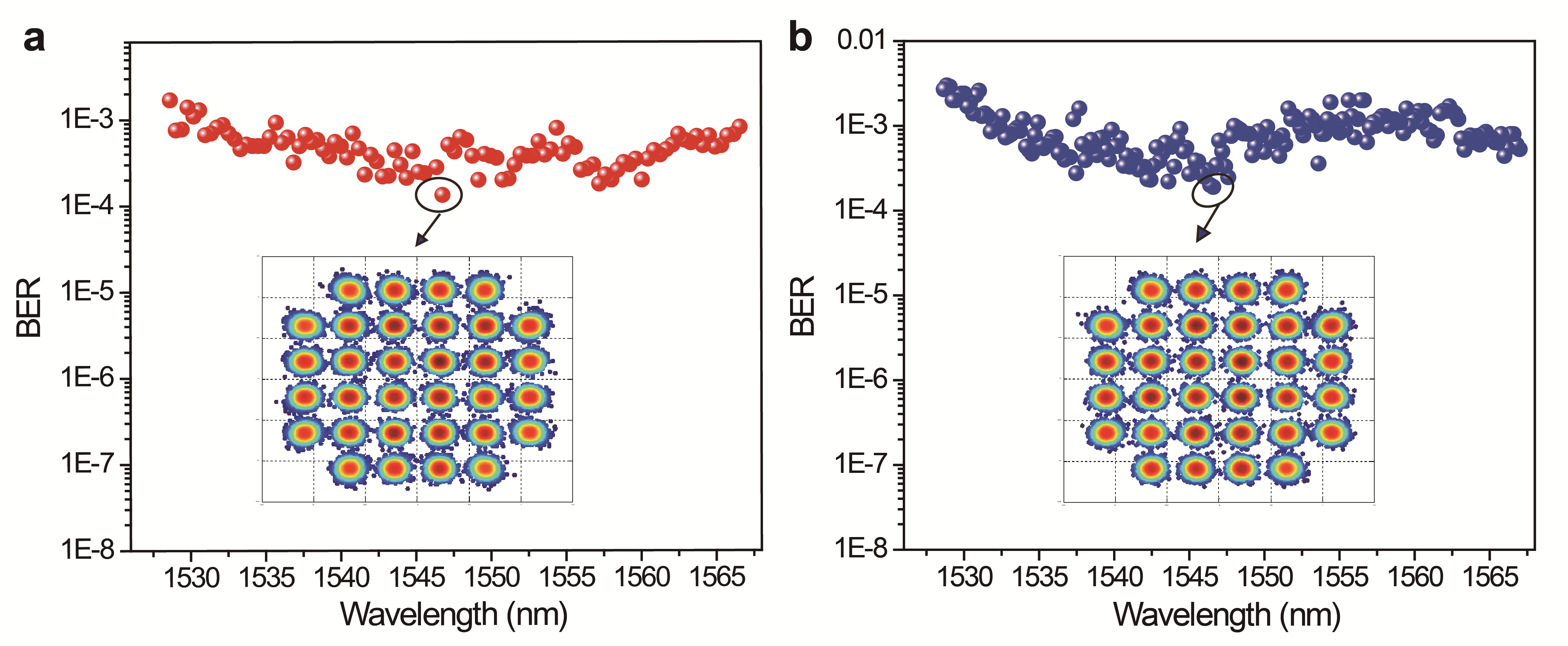


Figure S15 | 32QAM communication experiment results with wavelength scanning from 1527 to 1567 nm with a wavelength scanning interval of 0.2 nm. a, Bit error rate (BER) measurement for the +1 OAM mode generation. b, BER measurement for the +1 OAM mode detection.

To quantify the quality of the received signals, bit error rate estimations were performed. The results for the OAM emission and detection are shown in Fig. 4 in the main text.

References

1. Shen B, Wang P, Polson R, Menon R. An integrated-nanophotonics polarization beamsplitter with 2.4 × 2.4 μm2 footprint. *Nature Photonics* 2015, **9**(6)**:** 378-382.

2. Piggott AY, Lu J, Lagoudakis KG, Petykiewicz J, Babinec TM, Vučković J. Inverse design and demonstration of a compact and broadband on-chip wavelength demultiplexer. *Nature Photonics* 2015, **9**(6)**:** 374-377.
